# NMD is required for timely cell fate transitions by fine-tuning gene expression and regulating translation

**DOI:** 10.1101/gad.347690.120

**Published:** 2022-03-01

**Authors:** Michelle Huth, Laura Santini, Elena Galimberti, Julia Ramesmayer, Fabian Titz-Teixeira, Robert Sehlke, Michael Oberhuemer, Sarah Stummer, Veronika Herzog, Marius Garmhausen, Merrit Romeike, Anastasia Chugunova, Friederike Leesch, Laurenz Holcik, Klara Weipoltshammer, Andreas Lackner, Christian Schoefer, Arndt von Haeseler, Christa Buecker, Andrea Pauli, Stefan L. Ameres, Austin Smith, Andreas Beyer, Martin Leeb

**Affiliations:** 1 Max Perutz Laboratories Vienna, University of Vienna, Vienna BioCenter, 1030 Vienna, Austria;; 2 Vienna BioCenter PhD Program, Doctoral School of the University of Vienna, Medical University of Vienna, 1030 Vienna, Austria;; 3 Cluster of Excellence Cellular Stress Responses in Aging-Associated Diseases (CECAD), University of Cologne, 50931 Cologne, Germany;; 4 Institute of Molecular Biotechnology, Vienna BioCenter, 1030 Vienna, Austria;; 5 Research Institute of Molecular Pathology, Vienna BioCenter, 1030 Vienna, Austria;; 6 Center for Integrative Bioinformatics Vienna, Max Perutz Laboratories, University of Vienna, Medical University of Vienna, 1030 Vienna, Austria;; 7 Department for Cell and Developmental Biology, Medical University of Vienna, 1090 Vienna, Austria;; 8 Bioinformatics and Computational Biology, Faculty of Computer Science, University of Vienna, 1090 Vienna, Austria;; 9 Wellcome-MRC Cambridge Stem Cell Institute, University of Cambridge, Cambridge CB2 0AW, United Kingdom;; 10 Faculty of Medicine, University Hospital of Cologne, Center for Molecular Medicine Cologne, University of Cologne, 50937 Cologne, Germany;; 11 Institute for Genetics, Faculty of Mathematics and Natural Sciences, University of Cologne, 50923 Cologne, Germany

**Keywords:** embryonic stem cells, RNA biology, cell fate regulation, exit from naïve pluripotency, nonsense-mediated mRNA decay, pluripotency, translation initiation

## Abstract

Cell fate transitions depend on balanced rewiring of transcription and translation programs to mediate ordered developmental progression. Components of the nonsense-mediated mRNA decay (NMD) pathway have been implicated in regulating embryonic stem cell (ESC) differentiation, but the exact mechanism is unclear. Here we show that NMD controls expression levels of the translation initiation factor *Eif4a2* and its premature termination codon-encoding isoform (*Eif4a2*^*PTC*^). NMD deficiency leads to translation of the truncated eIF4A2^PTC^ protein. eIF4A2^PTC^ elicits increased mTORC1 activity and translation rates and causes differentiation delays. This establishes a previously unknown feedback loop between NMD and translation initiation. Furthermore, our results show a clear hierarchy in the severity of target deregulation and differentiation phenotypes between NMD effector KOs (*Smg5* KO > *Smg6* KO > *Smg7* KO), which highlights heterodimer-independent functions for SMG5 and SMG7. Together, our findings expose an intricate link between mRNA homeostasis and mTORC1 activity that must be maintained for normal dynamics of cell state transitions.

## Abstract (precis)

Here, Huth et al. investigated the role of components of the nonsense-mediated mRNA decay (NMD) pathway in regulating embryonic stem cell (ESC) differentiation, and show that NMD controls expression levels of the translation initiation factor *Eif4a2* and its premature termination codon-encoding isoform (*Eif4a2*^*PTC*^). Their findings expose an intricate link between mRNA homeostasis and mTORC1 activity that must be maintained for normal dynamics of cell state transitions.

Mouse embryonic stem cells (ESCs) capture the developmentally transient naïve pluripotent state in vitro. ESC self-renewal can be maintained in a naïve pluripotent state using inhibitors against Mek1/2 (PD0325901) and Gsk3 (CHIR990201; collectively termed “2i”). ESC self-renewal is sustained by an interactive transcription factor (TF) network, which has been extensively characterized (Young 2011; Dunn et al. 2014). Within 24–36 h after withdrawal of 2i, ESCs shut down naïve identity and initiate differentiation by transiting into formative pluripotency (Wray et al. 2011; Kalkan et al. 2017; Lackner et al. 2021). During this transition, the naïve gene regulatory network (GRN) is extinguished and expression of formative factors such as *Otx2*, *Pou3f1* (*Oct6*), *Dnmt3a/b*, and *Fgf5* is initiated. A similar transition is evident during peri-implantation development, where the TF network maintaining naïve pluripotency dissolves between embryonic day (E) 4.5 and E5.5 (Boroviak et al. 2014; Acampora et al. 2016; Mohammed et al. 2017).

High-resolution dissection of the exit from the naïve state is facilitated by the availability of Rex1 promoter-driven destabilized GFP reporter ESC lines (Rex1::GFPd2) (Kalkan et al. 2017; Mulas et al. 2017). Rex1 is a known marker of naïve pluripotency, and Rex1-GFPd2 down-regulation is initiated within 24 h after 2i withdrawal (N24) and completed after 48 h (N48). Extinction of Rex1-GFPd2 expression coincides with functional commitment to differentiation.

Several genome-wide screens have uncovered drivers of the exit from naïve pluripotency (Betschinger et al. 2013; Leeb et al. 2014; Li et al. 2020; Lackner et al. 2021), many of which are involved in transcriptional regulation and epigenetic modification. These screens also identified a large cohort of post-transcriptional regulators. RNA modifiers such as m^6^A (Batista et al. 2014; Geula et al. 2015), negative regulators of mRNA stability such as *Pum1* (Leeb et al. 2014), and components of the nonsense-mediated mRNA decay (NMD) pathway (Leeb et al. 2014; Li et al. 2015; Lou et al. 2016; Lackner et al. 2021) have been shown to play a role in ESC differentiation. Nonetheless, how post-transcriptional regulatory mechanisms contribute to cell fate changes remains poorly understood.

NMD is a translation-coupled mechanism that is crucial to maintain cellular RNA homeostasis by promoting degradation of potentially deleterious mRNAs containing a premature termination codon (PTC) (Kurosaki et al. 2019). However, PTC-independent NMD activity has also been shown (Mendell et al. 2004; Wittmann et al. 2006; Tani et al. 2012). NMD is triggered by phosphorylation of the RNA helicase UPF1. Phosphorylated UPF1 (p-UPF1) is essential for NMD and recruits NMD downstream effectors of two distinct RNA degradation pathways (Medghalchi et al. 2001). NMD-mediated mRNA degradation can occur either by the SMG6-mediated endonucleolytic cleavage pathway or via the exonucleolytic cleavage pathway, which is mediated by a SMG5–SMG7 heterodimer (Kurosaki et al. 2019). How division of labor between the two pathways is arranged remains elusive, but transcriptome-wide analysis demonstrated that both decay pathways have highly overlapping mRNA targets (Colombo et al. 2017). SMG5, SMG6, and SMG7 have also been shown to participate in recycling of NMD components by interacting with PP2A to mediate dephosphorylation of p-UPF1 (Anders et al. 2003; Chiu et al. 2003). Notably, recent data suggest that the endonucleolytic and exonucleolytic decay pathways are not acting in a strictly independent manner, and the SMG5–SMG7 heterodimer has been shown to play an important role in the SMG6-mediated endonucleolytic decay pathway (Boehm et al. 2021).

Although NMD components constitute some of the top hits in genome-wide screens studying exit from naïve pluripotency, neither the contribution of individual NMD effector proteins (SMG5, SMG6, and SMG7) nor the key mRNA targets of NMD that lead to a delayed cell fate transition are known.

Here we identify a role for NMD in ensuring normal differentiation kinetics by facilitating establishment of proper cell state-specific gene expression programs and by controlling expression of *Eif4a2*, a key translation initiation factor. We identify an *Eif4a2*-dependent increased mTORC1 activity in NMD-deficient ESCs as a factor contributing to their inability to properly commit to differentiation.

## Results

### Graded defects in exit from naïve pluripotency in NMD-deficient ESCs

**Figure 1.**
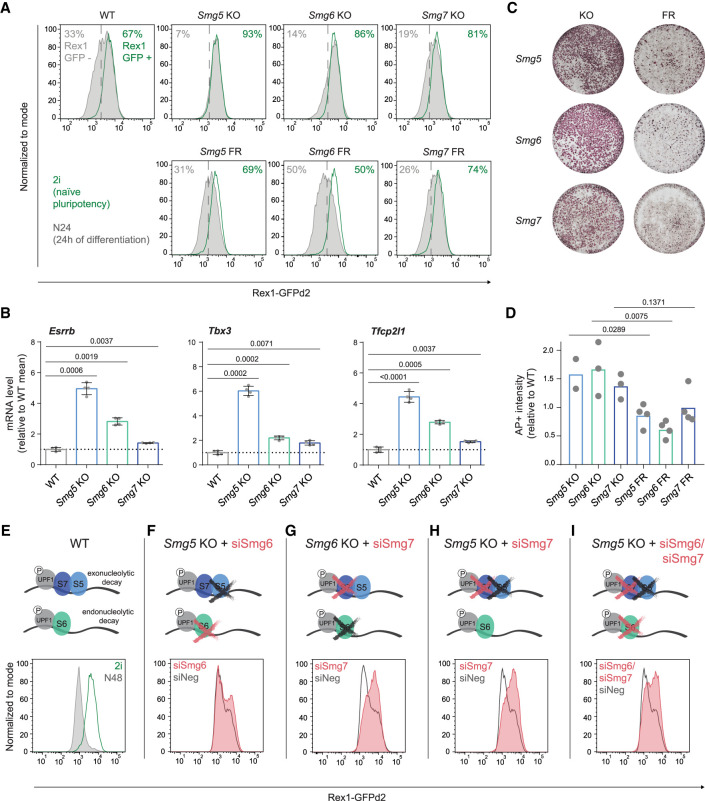
Hierarchy of defects in the exit from naïve pluripotency in NMD-deficient ESCs. (*A*) Rex1-GFPd2 analysis by flow cytometry in naïve pluripotency-supporting conditions (2i, green profiles) and 24 h after induction of differentiation by withdrawing 2i in N2B27 medium (N24, gray profiles) in WT, NMD KO, and NMD rescue ESCs (Flag rescue [FR]). The plots shown correspond to one experiment, representative for *n* = 5 independent replicates. (*B*) RT-qPCR analysis of the indicated naïve transcription factors (TFs) at N24 in WT and Smg factor KO ESCs. Mean and standard deviation (SD) are plotted for each cell line; *n* = 2 biological replicates. Expression was normalized to *β-actin*; results are shown as expression level relative to WT. Two-tailed *t*-tests were performed to compare WT with Smg factor KOs, and resulting *P*-values are indicated in the plot. (*C*) Commitment assay in NMD KO and NMD rescue ESCs. Alkaline phosphatase (AP) staining was used to visualize ESC colonies. (*D*) Quantification of commitment assays. Mean and SD are shown. Unpaired, two-tailed *t*-tests were performed to compare each Smg factor KO with its respective Flag rescue (FR) cell line. *P-*values are indicated in the plot. (*E*–*I***)** Rex1-GFPd2 flow cytometry analysis of WT cells (*E*) and Smg factor KO cells (*F*–*I*) at N48, after siRNA-mediated knockdown (KD) of *Smg6* or *Smg7*. Plots show one out of two independent experiments with consistent results.

To delineate the molecular function of NMD in the exit from naïve pluripotency, we generated Rex1-GFPd2 reporter ESC lines (Wray et al. 2011; Li et al. 2018) deficient for the three NMD downstream effectors *Smg5*, *Smg6*, or *Smg7* (NMD KO ESCs), as well as corresponding rescue cell lines in which the missing NMD factor was re-expressed (NMD Flag rescue ESCs [FR]) (Supplemental Fig. S1A–C). All Smg factor KO ESCs showed normal cell cycle and growth profiles (Supplemental Fig. S1D,E). However, they all exhibited pronounced delays in the exit from naïve pluripotency. This manifested in delayed down-regulation of the Rex1-GFPd2 reporter 24 h after the onset of differentiation (N24), and significant delays in the down-regulation of components of the core naïve transcription factor network (such as *Esrrb*, *Tbx3*, and *Tfcp2l1*) (Fig. 1A,B; Supplemental Fig. S1F). Notably, the three Smg factor KOs displayed variable degrees of impairment in exit from naïve pluripotency: The strongest effect was reproducibly observed in the absence of *Smg5* and the weakest was observed in the absence of *Smg7*.

Smg factors are known to regulate telomere maintenance (Reichenbach et al. 2003; Azzalin et al. 2007; Chawla and Azzalin 2008). However, no telomere shortening was observed in NMD KO ESCs, which exhibited either similar (*Smg6* KO) or even longer (*Smg5* and *Smg7* KOs) telomeres compared with wild-type (WT) ESCs (Supplemental Fig. S1G).

To examine later differentiation events, we assayed the ability of Smg factor KO ESCs to irreversibly exit the naïve state and commit to differentiation. We exposed them to differentiation conditions for 72 h and then reapplied 2i medium. Only cells that retain naïve identity are able to grow in 2i and form alkaline phosphatase (AP)-positive colonies. We observed a clear Smg factor KO-dependent delayed entry into commitment (Fig. 1C,D).

To monitor the effect of NMD deficiency on long-term differentiation potential, we performed 3D aggregate embryoid body (EB) differentiation throughout a 10-d time course (Keller et al. 1993). Down-regulation of naïve (*Esrrb*) and general (*Pou5f1*, also known as *Oct4*) pluripotency markers was severely impaired in NMD KO EBs (Supplemental Fig. S1H,I). Although Smg factor KO EBs up-regulated the formative marker genes *Fgf5* and *Oct6* with kinetics similar to those of WT, the subsequent shutdown of the formative program, initiated in WT cells around day 4 (d4), was impaired in NMD mutant cells. Furthermore, expression of the endo–mesoderm defining TF *Brachyury* (*T*) was not properly down-regulated after a peak of expression between d4 and d6, suggesting a general function of NMD in early cell fate decisions. In addition, teratomas derived from *Smg6* KO ESCs showed a lower degree of differentiation than WT and rescue controls (Supplemental Fig. S1J).

Together, these data show a global differentiation defect of Smg factor-deficient cells, with an unexpectedly variable and graded degree of phenotypic strengths (*Smg5* KO > *Smg6* KO > *Smg7* KO) during the exit from naïve pluripotency. This does not align with a proposed heterodimer-dependent function of SMG5 and SMG7, which would predict similar phenotypes for *Smg5* and *Smg7* KOs (Ohnishi et al. 2003; Okada-Katsuhata et al. 2012; Jonas et al. 2013). The results therefore suggest additional functions for SMG5 and SMG7 that are heterodimer-independent.

### Cooperativity between Smg factors regulates NMD function and the exit from naïve pluripotency

To investigate potential cooperativity between NMD effectors in regulating the exit from naïve pluripotency, we performed siRNA-mediated knockdowns of *Smg6* and *Smg7* in *Smg5* and *Smg6* KO ESCs (Fig. 1E–I; Supplemental Fig. S1K). Thereby, we generated cells double- and triple-depleted for the three NMD downstream effectors. WT cells showed a near-complete loss of Rex1-GFPd2 expression at 48 h of differentiation (N48) (Fig. 1E). siRNA-mediated depletion of *Smg6* in *Smg5* KO cells resulted in only a minor, nonsynergistic increase in the differentiation delay assessed in Rex1-GFPd2 reporter assays (Fig. 1F) despite the strong defects observed in both single KOs. In contrast, the depletion of *Smg7* in a *Smg6* KO background yielded a strong synergistic differentiation phenotype (Fig. 1G). Depletion of *Smg7* in *Smg5* KO ESCs also resulted in a clear synergistic effect (Fig. 1H), further highlighting heterodimer-independent functions for SMG5 and SMG7. Combined depletion of all the NMD effectors by double knockdown of *Smg6* and *Smg7* in *Smg5* KO ESCs resulted in differentiation delays on par with *Smg6/Smg7* or *Smg5/Smg7* double depletion (Fig. 1I). To assess the impact of NMD effector depletion and codepletion on NMD-specific mRNA target gene expression, we assessed expression levels of the known NMD target gene *Gadd45b* by RT-qPCR (Mendell et al. 2004). Consistent with observed differentiation delays, we observed the highest transcript levels of *Gadd45b* in *Smg6*/*Smg7* and *Smg5*/*Smg7* double-deficient cells. Double depletion of *Smg5* and *Smg6* resulted in a significantly weaker phenotype (Supplemental Fig. S1L). Together, this suggests that differentiation delays scale with the extent of NMD impairment.

To further study the cooperativity of NMD factors, we sought to generate stable NMD double-KO (dKO) ESCs. However, attempts using CRISPR–Cas9 to generate all possible dKO ESC lines yielded only *Smg5/Smg6* dKO ESCs. Neither *Smg5/Smg7* nor *Smg6/Smg7* dKOs could be established despite multiple attempts using efficient gRNAs (Supplemental Table S1). Although our inability to obtain *Smg5*/*Smg7* or *Smg6/Smg7* dKO ESCs does not rule out the feasibility of generating such dKO ESCs, our experiments indicate a strong counterselection against these double-mutant cell lines. This suggests that *Smg7* takes on a more important role for ESC self-renewal in the absence of its heterodimerization partner *Smg5* or in the absence of *Smg6.* This is also consistent with the exacerbated differentiation defects observed in *Smg5/Smg7* or *Smg6/Smg7* double-depletion experiments discussed above. *Smg5/Smg6* dKO cells showed a deficiency in down-regulating naïve TF mRNAs similar to that observed in *Smg5* single-KO cells, indicating an epistatic role of *Smg5* in regulating differentiation-relevant functions (Supplemental Fig. S1M).

Taken together, our results show that NMD effectors act synergistically during the exit from pluripotency. *Smg7* can be depleted without impacting the ESC state or strongly affecting early differentiation, but codepletion with either *Smg5* or *Smg6* results in apparently nonviable phenotypes. This suggests an important and potentially essential role for *Smg7* that is only revealed in the absence of either *Smg5* or *Smg6*.

### SMG7 alone is sufficient for p-UPF1 binding and is required for mediating the SMG5–p-UPF1 interaction

**Figure 2.**
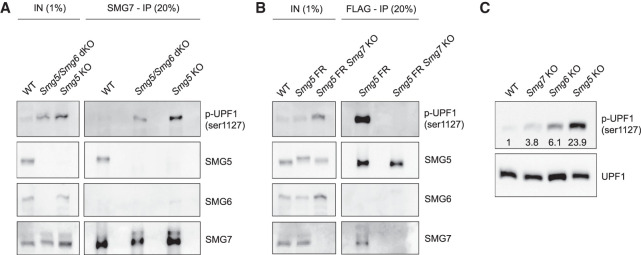
SMG7 alone is sufficient for p-UPF1 binding and is required for mediating the SMG5–p-UPF1 interaction. (*A*) Western blot analysis showing results of SMG7 co-IP in the indicated cell lines after cyanase-mediated nucleic acid removal. The antibodies used are indicated. (*B*) Western blot analysis of SMG5 co-IP (Flag antibody co-IP) in the indicated WT and *Smg5* Flag rescue (FR)-based cell lines after cyanase treatment. The antibodies used are indicated. *A* and *B* show one representative of two experiments with consistent results. (*C*) Western blot analysis for UPF1 and p-UPF1 expression in WT and NMD KO ESCs. Numbers in the plot are relative intensities compared with WT, normalized to total UPF1 levels.

To better understand the interplay between SMG5, SMG6, and SMG7 and to identify the molecular basis for an apparent heterodimer-independent function of SMG5 and SMG7, we performed coimmunoprecipitation (co-IP) experiments. We precipitated SMG5 or SMG7 in WT and NMD KO ESCs in the presence or absence of RNA. SMG5 and p-UPF1, but not SMG6, coimmunoprecipitated with SMG7 in an RNA-independent manner in WT ESCs, suggesting protein–protein complex formation (Fig. 2A; Supplemental Fig. S2A). Interestingly, SMG7 efficiently bound p-UPF1 in the absence of its heterodimerization partner SMG5. This SMG7–p-UPF1 interaction was also maintained in *Smg5/Smg6* dKO ESCs, suggesting that SMG7 can interact with p-UPF1 independently of other downstream NMD effectors. Conversely, however, SMG5 failed to bind p-UPF1 in the absence of SMG7 (Fig. 2B; Supplemental Fig. S2B), indicating that SMG7 provides the critical docking site for p-UPF1 during NMD and that p-UPF1 binding to SMG7 is independent of *Smg5*. When we performed the same co-IP experiments in the presence of RNA, we observed an interaction between SMG5 or SMG7 with SMG6 in the absence of the respective heterodimerization partner (Supplemental Fig. S2C,D). This RNA-dependent interaction was not observed in WT cells, suggesting aberrant cobinding of components of the endonucleolytic (SMG6) and exonucleolytic (SMG5 or SMG7) branches of NMD to the same RNA molecules in *Smg5* or *Smg7* KO ESCs, albeit without SMG6–SMG7 or SMG6–SMG5 protein–protein complex formation.

Continuous NMD activity relies on cyclic phosphorylation and dephosphorylation of UPF1. We observed increased p-UPF1 levels in all NMD KO cells (Fig. 2C), in line with previous reports (Okada-Katsuhata et al. 2012). Notably, p-UPF1 levels were elevated in all three Smg factor KOs and followed the same gradient as the differentiation phenotype and deregulation of the NMD target *Gadd45b*: Their increase was strongest in *Smg5* KOs and least pronounced in *Smg7* KOs. UPF1 is phosphorylated by the kinase SMG1 upon NMD target recognition (Huang et al. 2011; Yepiskoposyan et al. 2011). *Smg1* mRNA is itself an NMD target, but in contrast to p-UPF1, *Smg1* is up-regulated to a similar extent in all three NMD KO ESCs (Supplemental Fig. S2E; Colombo et al. 2017). Therefore, the graded increase of p-UPF1 levels in NMD KOs is unlikely to be a direct effect of increased *Smg1* expression, and impaired UPF1 dephosphorylation might underlie the elevated p-UPF1 levels in NMD mutant cells.

Taken together, this confirms that SMG5 and SMG7 form a heterodimer but suggests that they have additional, biochemically separable, and heterodimer-independent roles that result in distinct phenotypes upon *Smg5* or *Smg7* depletion.

### Integrating transcriptome-wide gene expression with mRNA half-life analysis identifies relevant NMD targets during the exit from naïve pluripotency

To uncover the molecular mode of action by which NMD regulates the exit from naïve pluripotency, we applied a combinatorial approach based on the following logic: An NMD target transcript causally relevant in the context of differentiation must show elevated transcript levels, following the same graded pattern as the differentiation phenotype (fold change [FC] vs. WT: *Smg5* KO > *Smg6* KO > *Smg7* KO) and must show a concomitant increased mRNA half-life.

**Figure 3.**
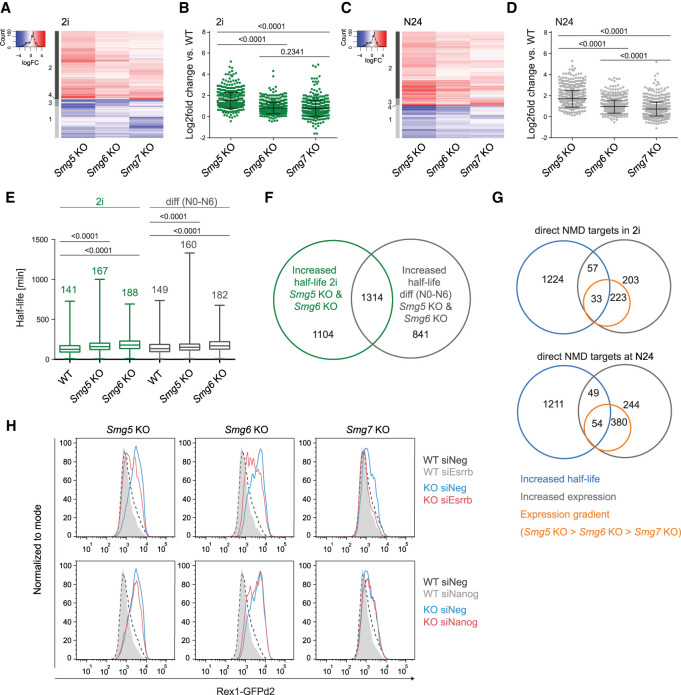
Integrating transcriptome-wide gene expression with mRNA half-life analysis identifies relevant NMD targets during the exit from naïve pluripotency. (*A*) Differentially expressed genes between WT and *Smg5*, *Smg6*, and *Smg7* KO ESCs in self-renewal (2i) (*P*adj ≤ 0.01; null hypothesis: |fold change (FC)| < 1.5). All genes fulfilling this cutoff in any of the three Smg factor KOs are shown. Clusters are indicated at the *left*. (*B*) Log_2_ fold change of deregulated transcripts in 2i belonging to clusters 2 and 4 (564 transcripts). Mean and SD are indicated in the graph. Paired, two-tailed *t*-test was used to calculate the indicated *P*-values. (*C*) Differentially expressed genes between WT and NMD KO ESCs at N24 (*P*adj ≤ 0.01; null hypothesis: |FC| < 1.5). All genes fulfilling this cutoff in any of the three Smg factor KOs are shown. (*D*) Log_2_ fold change of deregulated transcripts at N24 belonging to clusters 2 and 3 (808 transcripts). Mean and SD are indicated in the graph. Paired, two-tailed *t*-test was used to calculate *P*-values. (*E*) Half-life analysis in WT, *Smg5* KO, and *Smg6* KO ESCs in 2i and within the first 6 h after 2i withdrawal (N0–N6). Half-lives were calculated in minutes based on SLAM-seq experiments. Mean and SD are indicated in the graph. Paired *t*-test was used to calculate the indicated *P*-values. (*F*) Overlap between genes with a half-life (HL) increase of >10% in 2i and at the onset of differentiation (N0–N6) in both *Smg5* and *Smg6* KO cells. One-thousand-three-hundred-fourteen genes showed an increase in HL in all conditions. (*G*) Venn diagrams showing the overlap between the set of 1314 transcripts with increased HLs (blue), genes up-regulated in RNA-seq (gray), and genes up-regulated following the expression gradient (FC vs. WT: *Smg5* KO > *Smg6* KO > *Smg7* KO; orange) in 2i (*top* panel) or at N24 (*bottom* panel). (*H*) FACS plots showing the effects of siRNA-mediated depletion of *Esrrb* (*top*) or *Nanog* (*bottom*) on the differentiation delay exhibited by *Smg5*, *Smg6*, or *Smg7* KOs, measured by Rex1-GFPd2 flow cytometry analysis. WT profiles are shown in light gray (target siRNA) and in dashed black lines (negative control siRNA). Indicated mutant profiles are shown in blue (negative control siRNA) or red (target siRNA). One of three experiments with consistent results is shown.

To identify primary phenotypically relevant NMD targets, we first performed RNA-seq of *Smg5* KO, *Smg6* KO, *Smg7* KO, and WT cells in 2i and at N24 to assess the impact of NMD factor depletion on global gene expression (Supplemental Fig. S3A; Supplemental Table S2). In 2i, we identified 881 transcripts significantly deregulated in either of the Smg factor KO ESCs compared with WT (*P*adj ≤ 0.01), 516 of which were up-regulated and 252 of which were down-regulated in all three NMD KO ESCs (Fig. 3A). This shows a strong overlap in deregulated genes between all three Smg factor KO cell lines, further supporting the notion of largely overlapping target transcripts between SMG5, SMG6, and SMG7 (Colombo et al. 2017). Of the 516 transcripts up-regulated in all three KOs, 256 showed a graded increase in deregulation (FC vs. WT: *Smg5* KO > *Smg6* KO > *Smg7* KO) (Fig. 3B; Supplemental Table S2) without necessarily exhibiting significance in all pairwise comparisons between Smg factor KO ESCs. Gene ontology (GO) analysis of these 256 graded up-regulated transcripts in 2i revealed an enrichment for, e.g., methyltransferase and helicase activity (Supplemental Fig. S3B; Supplemental Table S3). The remaining up-regulated transcripts in 2i without a graded expression pattern in the KOs showed enrichment for, e.g., telomere maintenance and helicase activity. Within genes down-regulated in 2i, we detected a GO term enrichment for transcripts involved in neural development, somite development, and other differentiation-related processes, suggesting that NMD acts to destabilize naïve pluripotent cell identity already in the ESC state.

At N24, we identified 1174 significantly deregulated transcripts in Smg factor KO cells compared with WT cells (deregulated in either Smg factor KO; *P*adj ≤ 0.01) (Fig. 3C). Out of the 727 transcripts up-regulated in all NMD KO ESCs, 433 were up-regulated following a graded pattern as described above (FC vs. WT: *Smg5* KO > *Smg6* KO > *Smg7* KO) (Fig. 3D; Supplemental Table S2). GO analysis of these 433 transcripts revealed enrichment for pluripotency-related terms, such as LIF response and stem cell maintenance, which was not found within the nongraded up-regulated genes (Supplemental Fig. S3C; Supplemental Table S3). Within the graded down-regulated transcripts, we detected a specific enrichment of differentiation-related terms, such as neural tube development and pattern specification. Together, this suggests that genes showing graded expression at N24 are involved in and reflect the different levels of differentiation delays observed in NMD KO cells.

Previous reports showed increased *c-Myc* levels in NMD-deficient ESCs and proposed a causative involvement of *c-Myc* in NMD KO-induced differentiation delays (Li et al. 2015). In our data sets, we did not detect increased *c-Myc* transcript levels or observe increased protein levels in any of the three Smg factor KO ESCs (Supplemental Fig. S3D,E). On the contrary, we reproducibly detected reduced levels of c-MYC upon NMD disruption in ESCs. Furthermore, genetic depletion of *c-Myc* in Smg factor KO ESCs did not rescue their differentiation delays (Supplemental Fig. S3F,G), indicating that *c-Myc* is not causally involved in the naïve-to-formative transition defects observed in *Smg5*, *Smg6*, or *Smg7* KO ESCs. We attribute this discrepancy to the different, 2i-based culture conditions in our study that facilitated the identification of differentiation-relevant NMD targets, which are obscured in more heterogeneous FCS/LIF-based culture conditions.

Elevated transcript levels of direct NMD targets are expected to result from increased half-lives, based on impairment of mRNA degradation. We presumed that the NMD-responsive part of the machinery driving the extinction of the naïve network must show sensitivity to NMD depletion in 2i and/or at the onset of differentiation. We therefore chose to measure half-lives in 2i to identify factors potentially upstream of the naïve-to-formative differentiation cascade. We further performed half-life measurements between 0 and 6 h after 2i withdrawal (N0–N6) to detect the potential impact on the down-regulation of the naïve network because, during this time window, the sharpest drop of naïve TF mRNAs can be observed (Leeb et al. 2014; Lackner et al. 2021). Using SLAM-seq (Herzog et al. 2017), we were able to calculate half-lives for 4342 transcripts in 2i and 5250 transcripts at the onset of differentiation (N0–N6) in WT and *Smg5* and *Smg6* KOs (Supplemental Fig. S3H; Supplemental Table S4). We detected a global increase of transcript half-lives in *Smg5* and *Smg6* KO ESCs compared with WT ESCs at both time points. On average, mRNA half-life was significantly longer in NMD KOs than in WT, increasing from 141 to 167 and 188 min in 2i and from 149 to 160 and 182 min between N0 and N6 in WT and *Smg5* and *Smg6* KOs, respectively (*P* < 10^−4^ for all comparisons between KO and WT) (Fig. 3E). Overall, both the identity of transcripts and amplitude of half-life changes showed a strong overlap between *Smg5* and *Smg6* KO ESCs (Supplemental Fig. S3I,J). Transcriptome-wide comparisons showed that the vast majority of genes exhibiting elevated transcript levels showed a concomitant increase in half-lives (Supplemental Fig. S3K,L). Despite a relatively low sequencing depth, this allowed us to follow a step-wise filtering approach to stringently define a set of genes that showed a consistent increase in half-lives of >10% in four direct comparisons (2i: WT vs. *Smg5* KO, WT vs. *Smg6* KO; N0–N6: WT vs. Smg5 KO, WT vs. *Smg6* KO) (Fig. 3F; Supplemental Table S4), and within this set of genes to identify those that are of potential phenotypic relevance (graded expression pattern: FC vs. WT: *Smg5* KO > *Smg6* KO > *Smg7* KO). Thereby, we identified a set of 33 candidate genes in 2i and 54 candidate genes at N24. Twenty-four of those genes fulfil all criteria in both 2i and at N24 (Fig. 3G). We detected *Esrrb* as the only naïve transcription factor encoding mRNA within the group of 54 candidate NMD target transcripts with a graded expression at N24. This prompted us to probe whether the phenotype in Smg factor KO ESCs is dependent on increased levels of *Esrrb*. Indeed, siRNA-mediated depletion of *Esrrb* but not *Nanog* resulted in a partial rescue of Rex1-GFPd2 down-regulation kinetics (Fig. 3H), suggesting that increased half-life of *Esrrb* results in increased transcript levels at N24, which in turn causes a delay in the exit from naïve pluripotency. Bona fide NMD targets show elevated transcript levels after NMD inhibition achieved through translational inhibition by cycloheximide (CHX). *Esrrb* mRNA did not exhibit this hallmark feature of an NMD target and did not react to CHX treatment (Supplemental Fig. S4A), suggesting that although *Esrrb* up-regulation is causative for the Smg factor KO phenotype, other primary targets are directly regulated by NMD to ensure proper exit from the naïve pluripotent state. We propose that the increased half-life of *Esrrb* might be due to a potential deregulation of other, NMD-independent mRNA-degradation processes in Smg factor KO ESCs.

### Eif4a2 is a bona fide NMD target in ESCs, and its NMD sensitivity is evolutionarily conserved

**Figure 4.**
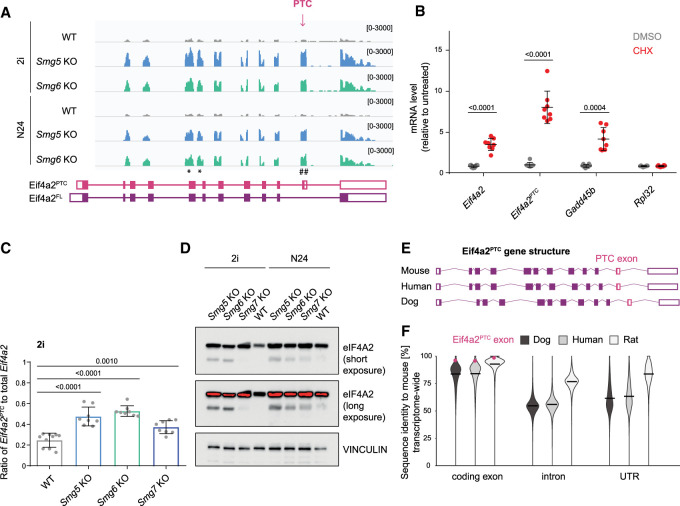
*Eif4a2* is a bona fide NMD target in ESCs. (*A*) Genome browser view of the *Eif4a2* locus visualizing RNA-seq results in WT, *Smg5* KO, and *Smg6* KO in 2i and at N24. (*) RT-qPCR primers amplifying all *Eif4a2* isoforms; (#) RT-qPCR primers amplifying the *Eif4a2*^*PTC*^ isoform. (*B*) Expression levels of the indicated genes after treatment with cycloheximide (CHX) or DMSO in WT ESCs, measured by RT-qPCR. Data shown are normalized to untreated WT ESCs. Mean and SD are plotted; *n* = 4 biological replicates. Unpaired, two-tailed *t*-test was performed between the DMSO and CHX comparison for each gene, and the resulting *P*-values are indicated in the plot. (*C*) Plots showing expression levels of *Eif4a2*^*PTC*^ as a fraction of total *Eif4a2* expression in WT and Smg factor KO ESCs, measured by RT-qPCR. Data from *n* = 4 independent experiments are shown. Unpaired, two-tailed *t*-test was performed to compare WT with Smg factor KOs. *P*-values are shown in the plot. (*D*) Western blot analysis for eIF4A2 protein levels in WT and NMD KO ESCs in self-renewal (2i) and at N24. Vinculin was used as a loading control. (*E*) Schematic representation of the *Eif4a2* gene structure in mice, humans, and dogs. (*F*) The percentage of transcriptome-wide sequence identity of the indicated genomic features (coding exon, intron, and untranslated region [UTR]) between mice, dogs, humans, and rats. Pink indicates the relative position of the *Eif4a2* PTC-containing exon, and black lines indicate the mean identity.

We reasoned that genes with functional relevance for the exit from naïve pluripotency defects of Smg factor KO ESCs will be found within the set of 24 candidate genes showing increased half-lives and graded expression in 2i and at the onset of differentiation (Supplemental Fig. S4B). We tested how many of these were bona fide NMD targets and displayed increased transcript levels upon treatment with CHX. Indeed, 14 out of 18 evaluated transcripts within this group showed significant increases in transcript levels after CHX treatment (Supplemental Fig. S4C). Among these, our attention was drawn to the RNA helicase *Eif4a2,* a component of the eIF4F translation initiation complex, which mediates 5′ cap recognition by the small ribosomal subunit (Yoder-Hill et al. 1993; Li et al. 2001). Several layers of evidence suggest *Eif4a2* as a bona fide and phenotypically relevant NMD target: First, *Eif4a2* is a highly expressed gene (FPKM in WT in 2i = 150), and its graded increase in both 2i and at N24 combined with a consistent increase in half-life indicates that it can be of primary relevance for the exit from naïve pluripotency delay in NMD-deficient ESCs (Supplemental Figs. S3M, S4D). Second, the *Eif4a2* locus encodes for two splice isoforms: one full-length protein (eIF4A2^FL^) and one predicted PTC-containing isoform (eIF4A2^PTC^) (Fig. 4A). Third, *Eif4a2* mRNA levels were significantly increased after translation inhibition by CHX treatment (Fig. 4B), similar to the known NMD target *Gadd45b*. *Eif4a2*^*PTC*^ showed an even more pronounced sensitivity to CHX treatment, indicating that both *Eif4a2*^*FL*^ and *Eif4a2*^*PTC*^ mRNAs are NMD targets. Graded *Eif4a2*^*PTC*^ up-regulation in NMD mutant cells in 2i and at N24 was confirmed by RT-qPCR validation (Supplemental Fig. S4E). Furthermore, *Eif4a2*^*PTC*^ up-regulation is directly caused by the absence of Smg factors and hence is rescued by re-expression of the missing NMD effector in the respective KO cells.

*Eif4a2*^*PTC*^ transcript levels in WT cells are ∼20% of total *Eif4a2* transcript (Fig. 4C). In *Smg5* and *Smg6* KO ESCs, levels rise to 50%60%. eIF4A2^FL^ protein levels were increased in Smg factor KO cells in 2i and at N24 (Fig. 4D; Supplemental Fig. S4F). The *Eif4a2*^*PTC*^ isoform produced a protein that was weakly detected by Western blot analysis in WT and NMD rescue cells (Fig. 4D; Supplemental Fig. S4H) and strongly increased in all three Smg factor KO ESCs, reaching up to 50% of full-length protein levels in *Smg5* and *Smg6* KO cells (Fig. 4D; Supplemental Fig. S4G). The up-regulation of *Eif4a2* was unique among eIF4F complex members, since the others, including the close homolog *Eif4a1*, showed neither increased expression (Supplemental Fig. S4D,I) nor significantly longer half-lives in Smg factor KOs (Supplemental Table S4), and did not react to CHX treatment (Supplemental Fig. S4J). Taken together, this identifies *Eif4a2* as a bona fide NMD target in ESCs and shows that NMD disruption leads to the production of a specific eIF4A2^PTC^ protein.

The linkage of NMD to *Eif4a2* expression appears to be conserved in evolution. The gene structure of *Eif4a2* is strikingly similar and the exact position of the PTC in the PTC-containing exon is identical between mice, dogs, and humans (Fig. 4E). In a transcriptome-wide comparison, the *Eif4a2* PTC exon showed high conservation at the nucleotide level despite being under no apparent selective pressure to maintain protein-coding potential (only two amino acids are encoded in this exon before the PTC) (Fig. 4F): Less than 5% of all exons and <3% of all untranslated regions (UTRs) showed higher conservation rates between mice and rats than the *Eif4a2*^*PTC*^ exon (Supplemental Fig. S4K). In the comparisons with humans and dogs, these percentages were even lower (3.4% and 1.1% in humans, 1.3% and 0.7% in dogs, respectively). Hence, the sequence identity of the *Eif4a2* PTC-containing exon and therefore the potential of *Eif4a2* to be regulated by NMD are features that appear to be conserved in mammalian evolution.

### *Eif4a2*^*PTC*^ up-regulation is causative for defects in exit from naïve pluripotency in *Smg5*-deficient ESCs

**Figure 5.**
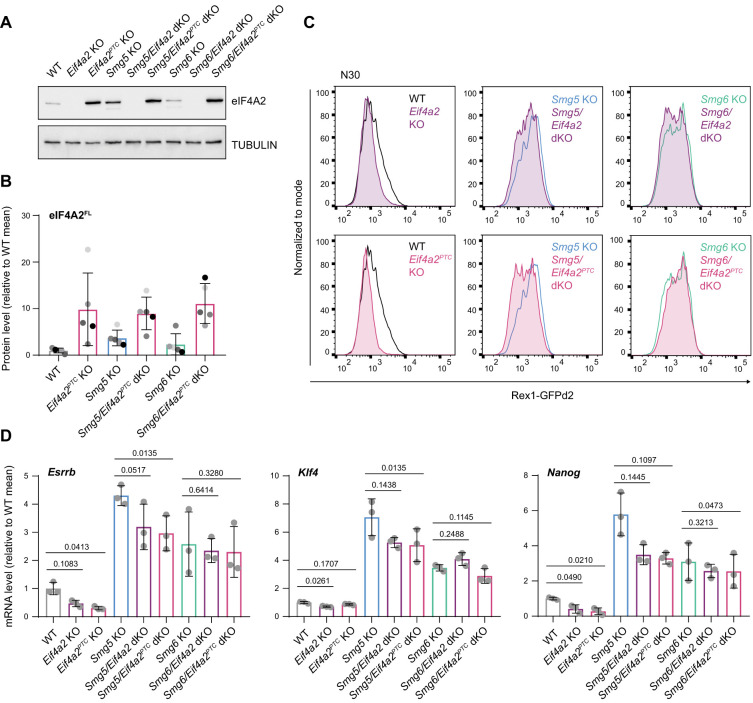
*Eif4a2* is causative for defects in exit from naïve pluripotency in *Smg5*-deficient ESCs. (*A*) Western blot analysis for eIF4A2 expression in the indicated cell lines. Tubulin was used as a loading control. (*B*) Quantification of eIF4A2^FL^ protein expression upon *Eif4a2*^*PTC*^ deletion in WT and NMD KO ESCs, measured by quantitative Western blot analysis. Expression levels were normalized to GAPDH; results are shown as expression levels relative to the WT mean. Mean and SD for *n* = 5 independent experiments are shown. (*C*) Rex1-GFPd2 flow cytometry analysis at N30 in WT, *Smg5* KO, and *Smg6* KO ESCs deleted for *Eif4a2* (purple) or *Eif4a2*^*PTC*^ (pink). Parental cell lines are shown for comparison. (*D*) RT-qPCR analysis of *Esrrb*, *Nanog*, and *Klf4* in the indicated cell lines. Expression was normalized to *Rpl32*; results are shown as expression levels relative to the WT mean. Mean and SD for *n* = 3 independent experiments are shown. A paired, two-tailed *t*-test was performed to compare each parental cell line with its respective *Eif4a2* or *Eif4a2*^*PTC*^ KO. *P-*values are indicated in the plot.

To delineate a potential causative relationship between increased *Eif4a2* levels and the differentiation defect observed in NMD-deficient cells, we generated *Eif4a2* KO cells and *Smg5*/*Eif4a2* or *Smg6*/*Eif4a2* double-deficient cells by deletion of either all potential *Eif4a2* isoforms (*Eif4a2* KO) or, specifically, *Eif4a2*^*PTC*^ (*Eif4a2*^*PTC*^ KO) (Fig. 5A; Supplemental Fig. S5A,B). Notably, deletion of the PTC exon resulted in a clear increase in full-length eIF4A2 protein levels in WT and NMD KO ESCs (Fig. 5B). Both the complete absence of *Eif4a2* and specific deletion of *Eif4a2*^*PTC*^ accelerated Rex1-GFPd2 down-regulation kinetics and down-regulation of naïve marker transcripts at N24 in WT cells (Fig. 5C,D). In *Smg5* mutants, deletion of *Eif4a2* or *Eif4a2*^*PTC*^ partially restored normal Rex1-GFPd2 down-regulation (Fig. 5C). Furthermore, transcript levels of *Esrrb*, *Klf4*, and *Nanog* were significantly reduced compared with *Smg5* single mutants at N30 (Fig. 5D), without, however, reaching WT levels, suggesting an incomplete rescue in *Smg5*/*Eif4a2* and *Smg5*/*Eif4a2*^*PTC*^ dKO cells. The differentiation defect observed in *Smg6* KO ESCs did not show a clear dependence on *Eif4a2*, suggesting that other mechanisms deregulated in *Smg6* KO ESCs are contributing to the differentiation phenotype to a larger extent than the up-regulation of *Eif4a2*^*PTC*^.

To further confirm the specificity of *Eif4a2* depletion in restoring differentiation potential in a *Smg5*-deficient ESC background, we compared the differentiation potential of *Tcf7l1* KO and *Eif4a2/Tcf7l1* dKO ESCs. *Tcf7l1* encodes for an HMG-box transcription factor and is a strong positive regulator of the exit from naïve pluripotency (Hoffman et al. 2013; Lackner et al. 2021). TCF7L1 is a transcriptional corepressor and acts downstream from the Wnt signaling cascade*. Tcf7l1* KO ESCs showed defects in exit from naïve pluripotency on par with NMD KOs but exhibited an NMD-independent, Gsk3 inhibition-like phenotype (Lackner et al. 2021). *Tcf7l1* KO cells were insensitive to codepletion of *Eif4a2* (Supplemental Fig. S5C,D), suggesting that *Eif4a2* is a specific genetic interactor of NMD in regulating differentiation.

In sum, the phenotypic rescue in *Smg5*/*Eif4a2*^*PTC*^ double-KO cells, in which eIF4A2^FL^ protein levels are even further increased, supports the proposition that *Eif4a2*^*PTC*^, rather than the full-length isoform, is a major mediator of the observed exit from naïve pluripotency defect, at least in *Smg5*-deficient ESCs.

To test the sufficiency of *Eif4a2* up-regulation to cause a differentiation delay, we overexpressed Flag-tagged *Eif4a2*^*FL*^ or *Eif4a2*^*PTC*^ in WT ESCs (Supplemental Fig. S5E). Indeed, at N24, *Eif4a2*^*PTC*^-overexpressing cells showed increased transcript levels for the naïve TFs *Klf4*, *Esrrb*, *Tfcp2l1*, and *Nanog* and increased protein levels for NANOG (Supplemental Fig. S5F–H). Cells overexpressing *Eif4a2*^*FL*^ exhibited a weaker effect and no up-regulation of NANOG protein levels (Supplemental Fig. S5H). This is in accord with an increase of *Eif4a2*^*PTC*^ rather than *Eif4a2*^*FL*^ having a major impact on the exit from naïve pluripotency. Notably, the differentiation delays elicited by increasing expression levels of *Eif4a2* isoforms did not reach the intensity observed in NMD KO ESCs, suggesting that factors in addition to *Eif4a2* contribute to the failure to properly shut down naïve pluripotency in NMD-deficient ESCs.

### *Eif4a2*-mediated differentiation delay is functionally linked to *Eif4a2*^*PTC*^-dependent regulation of mTORC1 activity and translation

To identify potential direct eIF4A2^FL^ and eIF4A2^PTC^ targets, we performed RIP-seq (Keene et al. 2006) using *Eif4a2* KO ESCs engineered to only express either Flag-tagged eIF4A2^PTC^ or eIF4A2^FL^ (*Eif4a2*^*PTC*^ FR and *Eif4a2*^*FL*^ FR) (Supplemental Fig. S6A). Expression levels of eIF4A2^FL^ closely matched endogenous levels and the PTC isoform was weakly detectable, similar to the levels in WT. As expected for a translation initiation factor, we identified non-protein-coding transcripts such as *Meg3*, *Rian*, and *Malat1* as depleted in Flag-eIF4A2^FL^ RIP samples, indicating the specificity of the assay (Supplemental Table S5). Out of 9812 expressed transcripts (CPM > 1 in all input samples), we detected 2601 transcripts bound by eIF4A2^FL^ significantly stronger than in empty vector (EV) control cells (*P*adj ≤ 0.05, log_2_FC > 0), which was confirmed by RT-qPCR on a selected set of candidates (Supplemental Fig. S6B). The vast majority of mRNAs showed a close correlation between expression level and eIF4A2^FL^ binding, as expected for a general translation initiation factor. Only 31 transcripts showed eIF4A2^FL^ enrichment significantly higher than expected based on transcript abundance. Among those was *Mtor*, the core component of both the mTORC1 and mTORC2 complexes (Supplemental Table S5). In contrast, RIP-seq of Flag-eIF4A2^PTC^ resulted in only minimal enrichments on RNA, possibly owing to the low protein levels of eIF4A2^PTC^ protein.

**Figure 6.**
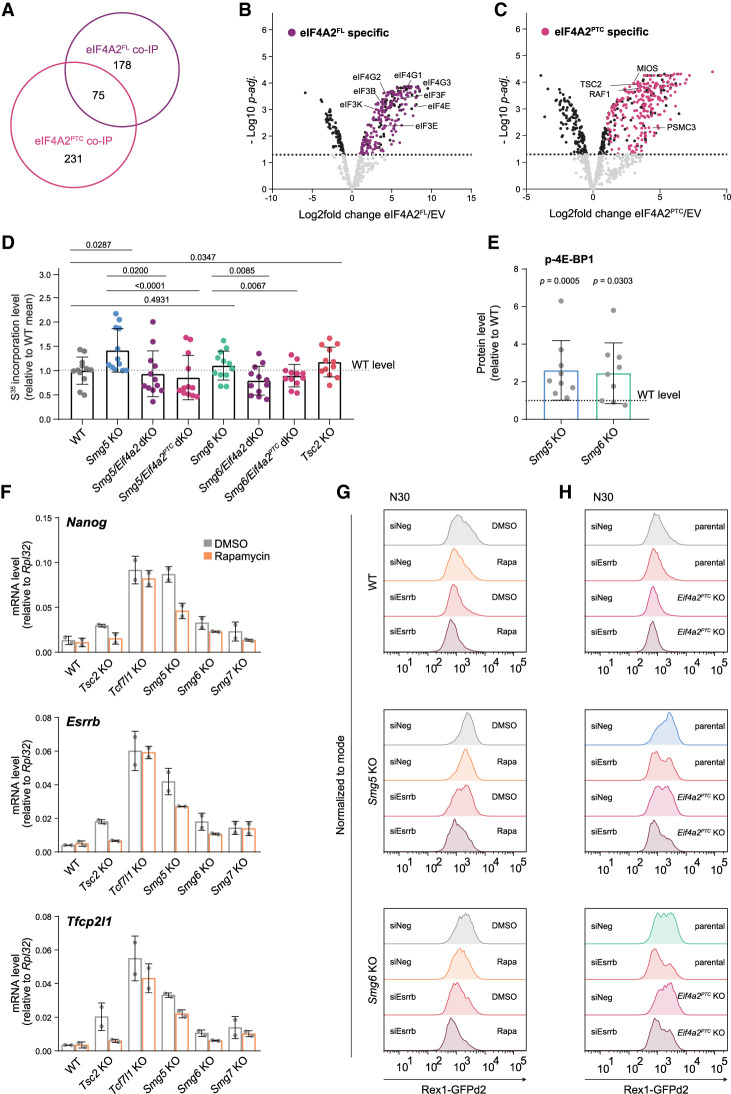
*Eif4a2*-mediated differentiation delay is caused by PTC isoform-dependent regulation of translation. (*A*) Venn diagram showing the overlap between eIF4A2^FL^- and eIF4A2^PTC^-bound proteins. Seventy-five proteins coprecipitate with both isoforms. (*B*) Volcano plot showing proteins detected by mass spectrometry after eIF4A2^FL^ coimmunoprecipitation. Black dots represent significantly enriched or depleted proteins (compared with empty vector [EV] co-IP), and purple dots represent proteins specifically enriched only in eIF4A2^FL^ co-IP (178 proteins; see *A*). (*C*) Volcano plot showing proteins detected by mass spectrometry after eIF4A2^PTC^ coimmunoprecipitation. Black dots represent significantly enriched or depleted proteins, and pink dots represent proteins specifically enriched only in eIF4A2^PTC^ co-IP (231 proteins; see *A*). (*D*) Translation initiation measured in ES-DMEM-2i medium by S^35^ incorporation into WT, Smg factor KO, Tsc2 KO, and Smg factor KO ESCs deficient for *Eif4a2* or *Eif4a2*^*PTC*^. Results are shown as incorporation levels relative to the WT mean. Mean and SD are plotted; *n* = 4 independent experiments with three biological replicates each. A two-tailed, paired *t*-test was used to calculate *P*-values. (*E*) Quantification of p-4E-BP1 protein expression in 2i in WT and Smg factor KO ESCs, measured by Western blot analysis. Expression was normalized to GAPDH; results are shown as expression levels relative to WT (dotted line). Mean and SD for *n* = 9 independent experiments are shown. Paired, two-tailed *t*-tests were performed to compare WT with Smg factor KO ESCs. *P*-values are indicated in the plot. (*F*) Expression levels of *Nanog*, *Esrrb*, and *Tfcp2l1* measured in WT, Smg factor KO, *Tsc2* KO, and *Tcf7l1* KO ESCs at N30 after treatment with DMSO or 20 nM rapamycin, measured by RT-qPCR. Expression levels are normalized to *Rpl32*. Mean and standard error of the mean (SEM) are shown; *n* = 2 independent experiments. (*G*) Rex1-GFPd2 analysis by flow cytometry at N30 in WT, *Smg5* KO, and *Smg6* KO ESCs transfected with control (siNeg) or siRNAs targeting *Esrrb* (siEsrrb) and treated with DMSO or 20 nM rapamycin (Rapa). (*H*) Rex1-GFPd2 profiles of WT, *Smg5* KO, and *Smg6* KO upon concomitant depletion of *Eif4a2*^*PTC*^ (*Eif4a2*^*PTC*^ KO) and siRNA-mediated *Esrrb* down-regulation (siEsrrb). Parental cell lines and control siRNA transfections (siNeg) are shown for comparison.

To understand the protein–protein interaction landscape of both eIF4A2 isoforms in ESCs, we performed co-IP followed by mass spectrometry. To compensate for the highly unstable nature of eIF4A2^PTC^, which is suggested by the discrepancy between *Eif4a2*^*PTC*^ transcript and eIF4A2^PTC^ protein levels relative to the respective full-length counterpart, we performed sample preparation after short-term treatment with the irreversible proteasome inhibitor epoxomicin (Supplemental Fig. S6C; Meng et al. 1999). Thereby, we identified 254 and 306 proteins specifically bound by eIF4A2^FL^ or eIF4A2^PTC^, respectively, over Flag-only control immunoprecipitation (IP) in EV cells (Fig. 6A; Supplemental Table S6).

Among the 178 proteins specifically bound to eIF4A2^FL^ and not to the PTC isoform were all eIF4G isoforms and several components of the eIF3 complex (Fig. 6B). This highlights the integration of eIF4A2^FL^ but not eIF4A2^PTC^ into a functional translation initiation complex.

Among the 75 proteins that coprecipitated with both isoforms, we identified several negative regulators of mRNA stability and regulation, such as AGO2, IGFBP1, IGFBP3, PUM1, TRIM71, CNOT, and the NMD components SMG6, SMG7, and UPF1 (Supplemental Fig. S6D). The interaction of eIF4A2 with SMG6 and SMG7 is consistent with previous reports (Schweingruber et al. 2016; Wilczynska et al. 2019). Together, this supports an intricate link between eIF4A2 and the mRNA destabilization machinery, which is potentially maintained in its PTC isoform. In addition, the GATOR complex member and mTOR regulator WDR59 precipitated with both isoforms (Supplemental Fig. S6D). eIF4A1 did not coprecipitate with either eIF4A2 isoform, suggesting that eIF4A1 and eIF4A2 participate in distinct, biochemically separable complexes.

Among the 231 proteins specifically associated with the PTC isoform and not the full-length protein, we detected a large cohort of chaperones, illustrating the unstable nature of eIF4A2^PTC^. However, we also detected interactions with the FGF/ERK pathway component RAF1, the GATOR complex member MIOS, and the key mTORC1 regulator TSC2 (Fig. 6C).

Taken together, these results show that eIF4A2^FL^ is mainly involved in translation initiation but also interacts with mRNA-destabilizing proteins, including NMD factors, CCR4–NOT components, and the RISC member AGO2. In contrast, eIF4A2^PTC^ protein showed little association with translation initiation complex members but still binds mRNA binding proteins and shows interaction with the negative mTORC1 pathway regulator TSC2.

eIF4A2 is part of the eIF4F complex, which integrates signals from the mTORC1 pathway to regulate translation levels (Sonenberg and Hinnebusch 2009). Together, the identification of several components of the mTORC1 complex and TSC2 as eIF4A2^FL^ and eIF4A2^PTC^ interactors and the detection of *Mtor* as a transcript enriched for eIF4A2 binding prompted us to ask whether increased levels of *Eif4a2* and *Eif4a2*^*PTC*^ in Smg factor KO cells impact on translational homeostasis in ESCs. To this end, we performed radioactive S^35^-based translation assays in *Smg5* or *Smg6* KO ESCs and corresponding *Eif4a2* and *Eif4a2*^*PTC*^ dKOs (Fig. 6D). We detected a significant *Eif4a2*^*PTC*^-dependent increase in translation rates in *Smg5* KO ESCs, similar in magnitude to that observed in *Tsc2* KOs, in which deregulated mTORC1 activity leads to an increase in translation (Grabole et al. 2016). Although increased translation rates in *Smg6* KO ESCs were not observed at a statistically significant level, we detected significant reductions of translation levels upon *Eif4a2*^*PTC*^ or *Eif4a2* depletion in *Smg6* KO ESCs. Because full-length eIF4A2 levels increase upon PTC isoform KO (Fig. 5B), these results permit the conclusion that increased translation rates in *Smg5* KO cells are mainly dependent on the PTC isoform of *Eif4a2*.

In line with a possible intersection of *Eif4a2* function with the mTOR pathway, increased mTORC1 activity was evident by significantly increased p-4E-BP1 levels in *Smg5* (*P* = 0.0005) and in *Smg6* (*P* = 0.0303) KO ESCs (Fig. 6E). Increased levels of p-4E-BP1 are dependent on eIF4A2^PTC^, as shown by a reduction of p-4E-BP1 levels upon codeletion of *Smg5* or *Smg6* with *Eif4a2*^*PTC*^ (Supplemental Fig. S6E). To determine the extent to which increased mTORC1 activity is causative for the observed differentiation defects in *Smg5*, *Smg6*, and *Smg7* KO ESCs, we treated differentiating cells with the mTORC1 inhibitor rapamycin (Betschinger et al. 2013; Villegas et al. 2019). As expected, in *Tsc2* KO ESCs, which exhibit an mTORC1-dependent phenotype, we detected a complete restoration of the down-regulation kinetics of the naïve markers *Nanog*, *Esrrb*, and *Tfcp2l1* as well as Rex1-GFPd2 levels upon rapamycin treatment, whereas the mTORC1-unrelated *Tcf7l1* mutants exhibited strong differentiation delays in both the presence and absence of rapamycin (Fig. 6F; Supplemental Fig. S6F–H). In contrast, rapamycin rescued differentiation defects of *Smg5*, *Smg6*, and *Smg7* KO ESCs, and mRNA levels of naïve TFs were clearly reduced upon rapamycin treatment in all Smg factor KO cells. Together, this shows that elevated mTORC1 activity is a key factor determining the differentiation delays of Smg factor-deficient ESCs. In *Smg5* KO ESCs treated with rapamycin, naïve TF expression was still clearly elevated compared with WT, indicating that deregulation of mTORC1-independent mechanisms also contributes to the delayed differentiation phenotype.

We therefore tested whether treatment of *Smg5* or *Smg6* KO cells with rapamycin together with concomitant destabilization of the naïve TF network is sufficient to restore normal differentiation kinetics. Indeed, simultaneous siRNA-mediated depletion of *Esrrb* with inhibition of mTORC1 activity resulted in WT-like Rex1-GFPd2 differentiation kinetics in both *Smg5* and *Smg6* KO cells (Fig. 6G). Similarly, deletion of *Eif4a2*^*PTC*^ combined with siRNA-mediated depletion of *Esrrb* could restore near-WT-like Rex1-GFPd2 down-regulation kinetics in Smg factor KO ESCs (Fig. 6H).

In summary, deregulation of a feedback circuit between NMD and translation initiation, encoded in a PTC-containing isoform of *Eif4a2*, results in elevated mTORC1 activity and increased translation rates in NMD-deficient ESCs. Although the relative contribution of *Eif4a2* to the translation state might differ between *Smg5* and *Smg6* KO ESCs, increased mTORC1 activity appears to be a crucial contributor to both KO phenotypes. We conclude that, in combination, the perturbance in RNA homeostasis, including increased levels of naïve pluripotency-specific transcripts, and the deregulation of translation through eIF4A2^PTC^ are the major contributors to NMD-associated differentiation defects in ESCs.

## Discussion

Depletion of *Smg5*, *Smg6*, or *Smg7* results in a graded impairment of NMD activity and causes corresponding delays in the exit from naïve pluripotency. Differentiation defects of *Smg5*, *Smg6*, and *Smg7* KOs scale with deregulation of known NMD target genes and levels of p-UPF1 (*Smg5* KO > *Smg6* KO > *Smg7* KO), indicating that NMD defects directly translate to differentiation delays at the exit from naïve pluripotency.

**Figure 7.**
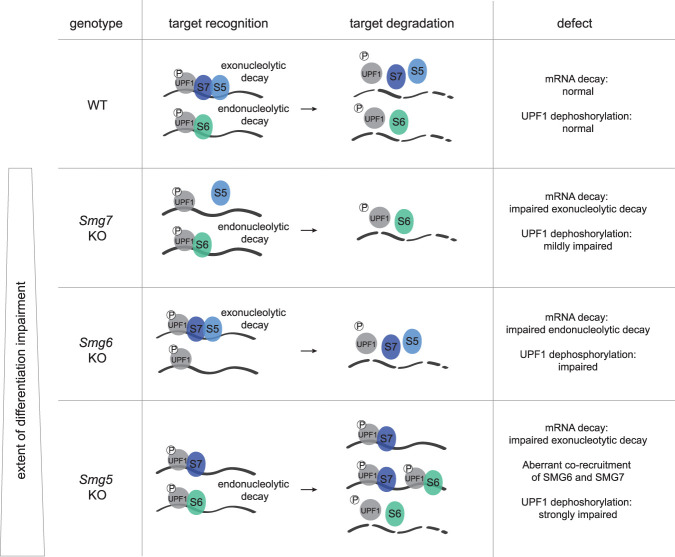
Schematic model of Smg factor activity resulting in a graded impairment of NMD and differentiation in *Smg5* KO, *Smg6* KO, and *Smg7* KO ESCs. Schematic illustration of the proposed interactions between NMD effectors and p-UPF1 dephosphorylation in WT, *Smg5* KO, *Smg6* KO, and *Smg7* KO ESCs that lead to graded defects in NMD and in the exit from naïve pluripotency.

The striking difference in NMD target regulation and extent of differentiation delays between *Smg5* and *Smg7* KOs (*Smg5* KO ≫ *Smg7* KO) together with the strong synergistic effects after codepletion are difficult to reconcile with the proposed obligatory heterodimer dependence of these two factors (Ohnishi et al. 2003; Jonas et al. 2013). Our data suggest a model in which a division of labor between SMG5 and SMG7 is causative for the different effects observed in the respective KOs (Fig. 7). Smg factors have a dual role in triggering RNA degradation and in mediating UPF1 dephosphorylation; p-UPF1 increase in all three Smg factor KOs is in accord with such a role of Smg factors in PP2A recruitment (Ohnishi et al. 2003; Okada-Katsuhata et al. 2012). Our data suggest that SMG7 acts as the main adapter or recruiter for p-UPF1, at least in naïve pluripotent ESCs. However, by itself it is unable to efficiently dephosphorylate UPF1, as evidenced by high p-UPF1 levels in *Smg5* KO ESCs, where SMG7 binding to p-UPF1 is unaffected. We propose that SMG7 binding to p-UPF1 without later UPF1 dephosphorylation by SMG5 results in jamming of the dephosphorylation cycle and the ensuing stalling of the mRNA degradation circuit in *Smg5* KO ESCs. Aberrant recruitment of SMG6 to mRNA targets already bound by SMG7 and p-UPF1 cannot properly restore NMD function in *Smg5* KOs. SMG5 alone in *Smg7* KOs is unable to bind p-UPF1. This suggests that the main role of SMG5 lies in its dephosphorylation activity, whereas recruitment to p-UPF1 is SMG7-dependent. This is consistent with the strongest increase of p-UPF1 levels and consequently the strongest differentiation delays in *Smg5* KO ESCs.

The relatively weaker phenotype in *Smg7* KOs is consistent with recent reports that SMG5 can compensate for the loss of SMG7 and that the presence of either SMG5 or SMG7 is sufficient to support SMG6-mediated endonucleolytic decay of NMD targets (Boehm et al. 2021). Notably, our observations of a weak *Smg7* KO phenotype without impact on cell proliferation and cell fitness are distinct from those recently made in HEK293 cells (Boehm et al. 2021) but are in accordance with the observation that *Smg6* depletion results in stronger NMD defects than *Smg7* deficiency (Colombo et al. 2017). It remains unclear whether the differences in cell fitness are due to distinct roles of *Smg7* between mice and humans or between ESCs, somatic cells, and established immortalized human cell lines.

Our data support a model in which SMG5 recruitment to p-UPF1-bound mRNAs is dependent on SMG7. Hence, in *Smg7* KO ESCs, the exonucleolytic decay pathway is completely inactive, but *Smg6* and the endonucleolytic pathway can largely compensate for loss of *Smg7* activity, resulting in only mild NMD defects in *Smg7* KO cells. However, SMG6 is unable to compensate for the complete absence of the SMG5–SMG7 heterodimer, suggested by the apparent loss of viability in a *Smg5/Smg7* dKO situation. This is consistent with the recent finding that the SMG5–SMG7 heterodimer is critical for NMD, even for SMG6-mediated endonucleolytic decay (Boehm et al. 2021), and our data further support an essential and partially redundant function of either SMG5 or SMG7 in the endonucleolytic decay pathway. Our results are consistent with this function being UPF1 dephosphorylation, which is also required in the SMG6-mediated endonucleolytic mRNA decay axis.

It is a conundrum that despite the weak defects in *Smg7* KO cells, facultative essentiality for *Smg7* is revealed in the absence of either *Smg5* or *Smg6*, and that *Smg7* alone, in the simultaneous absence of *Smg5* and *Smg6,* is sufficient to sustain minimal NMD activity required for survival. This suggests that SMG7 can either interact with potentially novel interactors in the absence of its normal interaction partners or act as a jack of all trades, fulfilling minimal roles in target recognition, recruitment of the degradation machinery, and UPF1 dephosphorylation. Taken together, the mode of action described above explains both the graded p-UPF1 levels and the graded impact on NMD of *Smg5*, *Smg6*, and *Smg7* KOs (Fig. 7). This model remains, in part, speculative but is fully in line with genetic and biochemical data.

We propose that the exit from naïve pluripotency defect in NMD mutant ESCs cannot be attributed to a single NMD target but results from a combination of strong impact on transcript levels with a major, not previously recognized influence of NMD on mTORC1 activity and the translation state of ESCs. Our data link the NMD-controlled *Eif4a2*^*PTC*^ genetically and biochemically to translation and mTORC1 activity. Consistent with our co-IP mass spectrometry results, a direct protein–protein interaction between eIF4A2 and TSC2 has been reported in *Drosophila* (Tsokanos et al. 2016); our data indicate that in mammalian cells, this interaction occurs between TSC2 and the eIF4A2^PTC^ isoform. Together, this supports the conclusion that NMD deficiency results in a dysbalanced translational state that is dependent on increased levels of eIF4A2^PTC^ protein and the likely resulting elevated mTORC1 activity. Control of protein synthesis rates is known to be fundamental for maintenance of self-renewal and differentiation (Bornelöv et al. 2019; Di Stefano et al. 2019) and for maintaining an ESC-specific chromatin state (Bulut-Karslioglu et al. 2018; Corsini et al. 2018). ESCs require down-regulation of translation rates and ribosome biogenesis to successfully differentiate (You et al. 2015; Corsini et al. 2018).

Although we can assign the main responsibility for the differentiation defect observed in NMD-deficient ESCs to increased mTORC1 activity and the NMD-responsive *Eif4a2*^*PTC*^ isoform, our data reveal a complex phenotype of NMD-deficient ESCs. The relative contribution of elevated *Eif4a2*^*PTC*^ to the overall differentiation defect differs between *Smg5* and *Smg6* KO cells. Whereas the differentiation defect of *Smg5* KO ESCs depends, to a large extent, on elevated *Eif4a2*^*PTC*^ expression, such dependence could not be detected in *Smg6* KOs. However, the deregulation of the naïve pluripotency circuitry in Smg factor KO ESCs was dependent on their significantly increased mTORC1 activity. This indicates that NMD controls mTORC1 activity and translation at various levels, not exclusively through regulation of *Eif4a2*^*PTC*^ expression.

We note that our mass spectrometry analysis detected the core pluripotency transcription factor proteins SOX2 and OCT4 as high-confidence interactors of eIF4A2^FL^ and eIF4A2^PTC^, respectively (Supplemental Table S6). However, without further experimentation, it cannot be ruled out that an interaction of cytoplasmic eIF4A2 with known nuclear factors is an artefact introduced after cell lysis before co-IP, although both OCT4 and SOX2 have been reported as RNA binding proteins before (Castello et al. 2012; He et al. 2016; Holmes et al. 2020).

NMD is triggered during translation. NMD-mediated regulation of *Eif4a2*, and specifically its PTC-containing isoform, provides a link back from NMD to translation initiation, thereby establishing a feedback circuit. We speculatively suggest that such a mechanism embedded in an NMD-responsive PTC-containing *Eif4a2* isoform can be used as a rheostat to balance NMD with translational activity. *Eif4a2* up-regulation upon NMD dysfunction increases translation initiation activity, which in turn directly increases the chances of triggering NMD in successive rounds of eIF4A2-initiated translation. Because *Eif4a1* is not subject to regulation by NMD, such a mechanism could also be used to shift the balance between levels and activity of eIF4A1 and eIF4A2. This is interesting because eIF4A1 and eIF4A2 have been proposed to have distinct cellular functions: Both are involved in cap-dependent translation initiation, but eIF4A2 appears also to play a role in translation inhibition through interaction with the CCR4–NOT complex (Meijer et al. 2013; Wilczynska et al. 2019). In line with the latter observation, our mass spectrometry data show interaction of both eIF4A2^FL^ and eIF4A2^PTC^ with several components of the CCR4–NOT complex and with AGO2.

These models remain speculation without further knowledge of the specific role of eIF4A2^PTC^ and its potential biochemical interaction with the translation initiation machinery and also with its full-length twin eIF4A2^FL^. Underscoring the importance of the *Eif4a2*^*PTC*^ isoform, it shows an unexpectedly high level of evolutionary conservation in various mammalian species, including humans. The exact function of eIF4A2^PTC^ and how it integrates into the translation machinery remain interesting questions for future research.

In conclusion, we show that maintenance of proper NMD activity is essential for restructuring of gene regulatory networks during the naïve-to-formative transition. In NMD-deficient cells, multiple transcripts, including naïve transcription factor mRNAs and most prominently those of the translation initiation factor *Eif4a2*, show increased transcription levels. Our results place NMD as a central player in shaping transcriptomes to maintain proper cellular identity. We provide evidence for an intricate feedback circuit between NMD and translation. NMD susceptibility is hardwired in an evolutionarily conserved fashion into the *Eif4a2* gene structure and sequence. This direct link between NMD and a translation initiation factor will need to be considered when studying NMD-associated phenotypes. Our results are also relevant for improving therapeutic interventions and provide a rationale for choosing the appropriate Smg factor in pharmacological approaches to inhibiting NMD activity, depending on the desired strength and extent of NMD inactivation (Kurosaki et al. 2019; Pawlicka et al. 2020).

## Materials and methods

### Cloning

gRNAs were designed using http://crisprscan.org. Annealed oligos were cloned in a gRNA-expressing vector (Addgene 41824) using a BsaI site (Mali et al. 2013; Lackner et al. 2021). gRNA sequences are indicated in Supplemental Table S7. For generating rescue cell lines, the coding sequence of the gene of interest was amplified by PCR and cloned into a pCAG-3xFlag-empty-pgk-hph vector (Betschinger et al. 2013).

### Teratoma assay

Paraffin-embedded teratoma tissue blocks were cut on a rotary microtome RM2255 (Leica). The 3-µm sections were then stained for hematoxylin and eosin in the automated slide stainer Gemini AS (Histocom) and mounted in Eukitt. Slides were scanned on a VS120 (Olympus) slide scanner.

### Cell culture

Mouse embryonic stem cells (ESCs) containing a Rex1-GFPd2-IRES-BSD reporter construct carrying a Cas9 transgene (RC9) (Wray et al. 2011; Li et al. 2018) were used as a parental cell line for all of the knockout cells generated in this study. A list of the primers used to perform genotyping PCRs in the derived KO cell lines is in Supplemental Table S7. ESCs were cultured on 0.2% gelatin-coated (Sigma-Aldrich G1890) plates in DMEM high-glucose (Sigma-Aldrich D5671) supplemented with 15% FBS (Gibco 10270-106), 2 mM L-glutamine (Sigma-Aldrich G7513), 0.1 mM NEAA (Sigma-Aldrich M7145), 1 mM sodium pyruvate (Sigma-Aldrich S8636), 10 µg/mL penicillin–streptomycin (Sigma-Aldrich P4333), 55 µM β-mercaptoethanol (Fisher Scientific 21985-023), 2i (1.5 μM CHIR99021 and 0.5 μM PD0325901), and 10 ng/mL LIF (batch tested, in-house; ES-DMEM + 2i medium). All cell lines were regularly negatively tested for mycoplasma infection.

### Monolayer differentiation

For differentiation, ESCs were plated on gelatin-coated plates at a density of 1 × 10^4^ cells/cm^2^ in basal medium (N2B27) composed of a 1:1 mix of DMEM/F12 (Gibco 21331020) and neurobasal medium (Gibco 21103049) supplemented with N2 (homemade), B27 serum-free supplement (Gibco 17504-044), 2 mM L-glutamine (Sigma-Aldrich G7513), 0.1 mM NEAA (Sigma-Aldrich M7145), 10 µg/mL penicillin–streptomycin (Sigma-Aldrich P4333), 55 µM β-mercaptoethanol (Fisher Scientific 21985-023), and 2i (3 μM CHIR99021 and 1 μM PD0325901; N2B27 + 2i medium). The following day, N2B27 + 2i medium was withdrawn, and cells were washed once with PBS and then differentiated for the indicated time in N2B27.

### Rapamycin treatment

ESCs were plated on gelatin-coated plates in N2B27 + 2i + DMSO (Sigma-Aldrich D2650) or N2B27 + 2i + 20 nM rapamycin (Enzo Life Sciences BML-A275-0005). The following day, N2B27 + 2i medium was removed and cells were differentiated in N2B27 + DMSO or N2B27 + 20 nM rapamycin for the indicated time after one wash with PBS.

### Commitment assay

ESCs were plated on gelatin-coated plates in N2B27 + 2i medium at a density of 2 × 10^3^ cells/cm^2^. The following day, N2B27 + 2i medium was withdrawn and cells were differentiated for 3 d in N2B27. After 72 h, medium was changed to N2B27 + 2i + blasticidin (BSD, Invivogen), and medium was refreshed every 2 d. Alkaline phosphatase staining was performed after 4 d in N2B27 + 2i + BSD (Sigma-Aldrich 86R). Plates were then imaged using an Olympus Cell-Sense microscope (Olympus).

### EB differentiation assay

EBs were formed as hanging drops in ESC culture medium without LIF and 2i (200 cells/20 µL drop). After 2 d, EBs were collected in a 10-cm Petri dish; medium was changed every 2 d. Cells were harvested every 2 d and RNA was extracted using the ExtractMe kit (LabConsulting EM09).

### Generation of knockout cell lines

Cells (2 × 10^5^) were reverse-transfected in a gelatin-coated six-well plate in ES-DMEM + 2i using Lipofectamine 2000 (Fisher Scientific 11668-027). Two gRNAs were used for each gene (1 µg of each) together with 0.5 µg of pCAG-dsRed. dsRED/GFP-double-positive cells were sorted using a BD FACS Aria III flow cytometer. Sorted cells were plated at clonal density in ES-DMEM + 2i and single-cell-derived colonies were manually picked 1 wk after sorting. Colonies were then trypsinized, and half of the cell suspension was plated in a 96-well plate for expansion. The remaining cells were lysed and DNA was extracted for PCR genotyping (Lackner et al. 2021). Clonal rescue cell lines were generated using piggyBac-based transgenesis. Clones with stable expression of rescue transgenes were isolated and used for further analysis. A correct karyotype (modal average of 40 chromosomes) was confirmed for all ESC lines, and cultures were regularly tested negative for mycoplasma infection.

### RNAi assay

In a gelatin-coated 12-well plate, 1.5 × 10^4^ cells/cm^2^ were transfected in N2B27 + 2i using DharmaFECT 1 (Fisher Scientific T-2001). For RNAi assays, esiRNA for *Esrrb* and *Nanog* (Sigma-Aldrich) and FlexiTube siRNAs for *Smg6* and *Smg7* (Qiagen) were used at a final concentration of 20 nM. The following day, medium was changed to N2B27 after two PBS washes and cells were differentiated for the indicated time. For the *Esrrb* RNAi assay combined with rapamycin treatment, 1 × 10^4^ cells/cm were transfected with 200 ng of esiRNA targeting *Esrrb* (Sigma-Aldrich EMU015811) in N2B27 + 2i medium. The day after, cells were treated for 4 h with either DMSO or 20 nM rapamycin in N2B27 + 2i medium before being differentiated in N2B27 + DMSO or N2B27 + 20 nM rapamycin for the indicated time. A PBS wash was performed after the removal of 2i, before switching to differentiation medium.

### Cell cycle analysis

ESCs were plated in gelatin-coated plates in ES-DMEM + 2i at a density of 1 × 10^4^ cells/cm^2^. After 2 d, cells were harvested and fixed in 70% ethanol. Cells were then incubated with 100 μg/mL RNase A solution (Qiagen 19101). Propidium iodide solution (50 mg/L; Sigma-Aldrich P4170) was added to the cells. Cell cycle profiles were recorded on a LSRFortessa flow cytometer (BD bioscience).

### Flow cytometry

Cells were harvested using 0.25% trypsin/EDTA, and trypsin was neutralized using ES-DMEM. Rex1-GFPd2 expression levels were measured with a LSRFortessa flow cytometer (BD bioscience). High-throughput measurements were acquired using a 96-well plate HTS unit. Data analysis was performed using FlowJo software (BD Bioscience).

### Translation inhibition assay

ESCs were plated in gelatin-coated plates in ES-DMEM-2i at a density of 2 × 10^4^ cells/cm^2^. The following day, cells were treated for 8 h with 100 µg/mL either DMSO or cycloheximide (CHX; Sigma-Aldrich 01810) and then harvested for RNA extraction.

### Telomere quantification

For telomere quantification, genomic DNA was extracted from cells using the Puregene core kit A (Qiagen). DNA was quantified using PicoGreen assay for dsDNA (Fisher Scientific P11496) on a NanoDrop 3300 Fluorospectrometer (Fisher Scientific). PCR reactions were performed on a CFX384 Touch real-time PCR detection system (Bio-Rad) using telomere-specific primers and primers for the single-copy gene 36B4. For each primer pair, a standard curve was created with known amounts of DNA to determine primer efficiency.

### RNA analysis

RNA was extracted using the ExtractMe kit (LabConsulting EM09) according to the manufacturer's protocol. cDNA was generated from 0.3–1 µg of RNA using the SensiFAST cDNA synthesis kit (Bioline BIO-65054). Real-time PCR was performed on the CFX384 Touch real-time PCR detection system (Bio-Rad) using the Sensifast SYBR no Rox kit (Bioline BIO-98020). All RT-qPCR primers used in this study are listed in Supplemental Table S7.

### Protein analysis

Proteins were extracted using RIPA buffer (Sigma-Aldrich 20-188) supplemented with Complete Mini EDTA-free protease inhibitor cocktail (Roche 04693159001) and PhosSTOP (phosphatase inhibitor cocktail [Roche 04906845001]). Five percent BSA was used for blocking for phospho antibodies, and 5% milk was used for all other antibodies. Primary antibodies were incubated overnight at 4°C or for 1 h at room temperature. Washes were performed using PBS-T (Sigma-Aldrich P4417). Secondary antibodies were incubated for 1 h at room temperature. Primary antibodies were used at a dilution of 1:250 for anti-SMG5 (rabbit; Abcam ab33033), 1:1000 for anti-SMG6 (rabbit; Abcam ab87539), 1:2000 for anti SMG7 (rabbit; NovusBio NBP1-22967), 1:1000 for anti-p-UPF1 (rabbit; Millipore 07-1016), 1:1000 for anti-UPF1 (D15G6; rabbit; Cell Signaling 12040S), 1:1000 for anti-Flag M2 (mouse; Sigma-Aldrich F1804), 1:1000 for anti-c-MYC (D84C12; rabbit; Cell Signaling 5605T), 1:1000 for anti-TCF7L1 (rabbit; Fisher Scientific PA5-40327), 1:1000 for anti-eIF4A2 (rabbit; Abcam ab31218), 1:1000 for anti-eIF4A1 (rabbit; Cell Signaling 2490T), 1:1000 for anti-NANOG (rabbit; NovusBio NB100-58842), 1:1000 for anti-p-4E-BP1(Ser65; rabbit; Cell Signaling 9451T), 1:5000 for anti-Tubulin (mouse; Sigma-Aldrich T8203), 1:5000 for anti-GAPDH (mouse; Sigma-Aldrich G8795), and 1:5000 for anti-Vinculin (E1E9V; rabbit; Cell Signaling 13901T). Secondary antibodies were used at a dilution of 1:10,000 for antirabbit IgG (Amersham NA934) and 1:15,000 for goat antimouse IgG (Santa Cruz Biotechnology sc-2064). Chemiluminescence signal from antibody binding was detected using ECL Select detection kit (GE Healthcare GERPN2235) with a ChemiDoc system (Bio-Rad). Western quantification was performed using ImageJ or ImageLab.

### Immunoprecipitation

Cells were plated in ES-DMEM + 2i, and after 2 d were harvested in IP lysis buffer (10 mM Tris Base, 10 mM NaCl, 2 mM EDTA, 0.5% Triton X-100) supplemented with Complete Mini EDTA-free protease inhibitor cocktail (Roche 04693159001) and PhosSTOP (phosphatase inhibitor cocktail [Roche 04906845001]). One milligram of lysate was used for the immunoprecipitation. Briefly, Dynabeads (Fisher Scientific 10004D) were coated with 5 µg of anti-Flag M2 antibody (mouse; Sigma-Aldrich F1804) for 1 h. For RNA-free IP, lysates were treated with 50 U/mL cyanase (Süd-Laborbedarf SLG CY1000) and 0.5 µL/mL 2 M MnSO_4_ and incubated for 10 min on ice. Lysates were cleared by centrifugation at 16,000*g* for 15 min at 4°C, and 10% of each lysate was kept as input. NaCl was added to a final concentration of 150 mM. Dynabeads were washed three times with wash buffer (137 mM NaCl, 20 mM Tris base, 0.5% [v/v] Tergitol-type NP-40) and one time with lysis buffer before incubation with the lysates for 3 h at 4°C. Dynabeads were washed three times with wash buffer supplemented with Complete Mini EDTA-free protease inhibitor cocktail (Roche 04693159001) and PhosSTOP (phosphatase inhibitor cocktail [Roche 04906845001]). Samples were eluted in 2× NuPAGE sample buffer.

For the immunoprecipitation coupled with mass spectrometry, cells were plated in ES-DMEM + 2i. The following day, they were treated with 1 µM epoxomicin (Gentaur 607-A2606) for 3 h. Cells were harvested and lysed in IP lysis buffer. One milligram of lysate was used for the immunoprecipitation. Immunoprecipitation protocol was followed (see above) with the addition of the cross-linking of the anti-Flag M2 antibody to the Dynabeads using dimethyl pimelimidate (DMP) (Sigma-Aldrich D-8388). Briefly, after antibody coupling, Dynabeads were washed three times with 200 mM sodium borate (pH 9) and then incubated for 30 min with DMP–sodium borate solution. They were washed with the following buffers: three times with 250 mM Tris (pH 8.0), twice with 100 mM glycine (pH 2), three times with TBS-T, and once with lysis buffer. Beads were then incubated with the lysates for 2 h at 4°C. Five washes were performed with wash buffer (137 mM NaCl, 20 mM Tris base).

### Sample preparation for mass spectrometry analysis

Beads with cross-linked antibodies were transferred to new tubes and resuspended in 30 µL of 2 M urea in 50 mM ammonium bicarbonate (ABC). Disulfide bonds were reduced with 10 mM dithiothreitol for 30 min at room temperature before adding 25 mM iodoacetamide and incubating for 15 min at room temperature in the dark. The remaining iodoacetamide was quenched by adding 5 mM DTT, and the proteins were digested with 150 ng of trypsin (Trypsin Gold, Promega) for 90 min at room temperature. The supernatant was transferred to a new tube, the beads were washed with another 30 µL of 2 M urea in 50 mM ABC, and the wash was combined with the supernatant. After diluting to 1 M urea with 50 mM ABC, an additional 150 ng of trypsin was added and incubated overnight at 37°C in the dark. The digest was stopped by addition of trifluoroacetic acid (TFA) to a final concentration of 0.5%, and the peptides were desalted using C18 Stagetips (Rappsilber et al. 2007). Peptides were separated on an Ultimate 3000 RSLC nano-flow chromatography system (Thermo Fisher) using a precolumn for sample loading (Acclaim PepMap C18, 2 cm long × 0.1-mm diameter, 5-μm particle size, Thermo Fisher) and a C18 analytical column (Acclaim PepMap C18, 50 cm long × 0.75-mm diameter, 2-μm particle size, Thermo Fisher), applying a segmented linear gradient from 2% to 35% and finally 80% solvent B (80% acetonitrile, 0.1% formic acid; solvent A: 0.1% formic acid) at a flow rate of 230 nL/min over 120 min. Eluting peptides were analyzed on a Q Exactive HF-X Orbitrap mass spectrometer (Thermo Fisher), which was coupled to the column with a customized nano-spray Easy-Spray ion source (Thermo Fisher) using coated emitter tips (New Objective).

### mRNA half-life measurement

ESCs were plated in N2B27 + 2i at a density of 2 × 10^4^ cells/cm^2^. The day after, the medium was changed to N2B27 + 2i + 100 µM 4SU (Carbosynth NT06186) and cells were cultured in this condition for 12 h. After a PBS wash, cells were incubated with N2B27 or N2B27 + 2i medium + 10 mM uridine (Sigma-Aldrich U6381) for 3 and 6 h. RNA extraction was carried out using Trizol (Fisher Scientific 10296-010) following the manufacturer's instruction with the addition of 0.1 mM DTT during the isopropanol precipitation. RNA was resuspended in 1 mM DTT. Five micrograms of RNA was treated with 10 mM iodoacetamide (Sigma-Aldrich I1149) followed by ethanol precipitation (Herzog et al. 2017). Two nanograms of RNA was used for library preparation. Libraries were prepared using the QuantSeq 3′mRNA-seq library preparation kit for Illumina FWD (Lexogen R3142) and were analyzed on a HiSeqV4 SR100.

### RNA immunoprecipitation

Cells (3 × 10^6^) were plated in ES-DMEM + 2i. After 2 d, cells were harvested and lysed using the Magna-RIP kit (Millipore 17-700) according to the manufacturer's protocol. Briefly, cells were lysed by a freeze and thaw cycle and lysates were stored at −80°C. Immunoprecipitations were performed for 3 h at 4°C using 5 µg of an anti-Flag M2 antibody (mouse; Sigma-Aldrich F1804). For RIP-seq, libraries were prepared using the QuantSeq 3′mRNA-seq library preparation kit for Illumina FWD (Lexogen R3142) and analyzed on a HiSeqV4 SR50. Relative binding to input and empty vector control was calculated.

### Translation rate measurement using S^35^ incorporation

ESCs were cultured in ES-DMEM + 2i and 100 µCi S^35^ (Hartmann IS-103) was added to the culture medium for 30 min (Yamasaki et al. 2009). Cells were washed twice in PBS and lysed in RIPA buffer (Sigma-Aldrich 20-188) supplemented with Complete Mini EDTA-free protease inhibitor cocktail (Roche 04693159001) as described above. Proteins were acetone-precipitated and resuspended in RIPA buffer after two rounds of acetone washes. Protein measurements were performed using a micro BCA protein assay kit (Thermo Fisher 23235). Seven microliters of protein extracts was then spotted on a nitrocellulose membrane and exposed to a BAS storage phosphor screen (GE Healthcare). After 6–12 h, the signal was acquired using a Typhoon scanner (GE Healthcare). Radioactive signal was quantified using the ImageQuant software.

### RNA-seq differential analysis

RNA-seq data and analysis used in this study were obtained from a previous study (Lackner et al. 2021). In brief, quality control was performed using FastQC (version 0.11.5). Transcripts were mapped to the mm10 mouse reference genome and counts were determined using STAR (version 2.5.3). RPKM values were calculated using DESeq2 (version 1.20.0). Differential expression analysis was carried out using limma (version 3.30.13). *P*-values were corrected for multiple testing using the Benjamini–Hochberg method (FDR). Clustering was performed using all genes significantly regulated (*P*adj < 0.01) in at least one of the Smg factor mutant cells using hclust. Cluster numbers are indicated in Figure 3, A and C. For RNA-seq, we used two independently derived KO cell lines for each of the Smg factors.

### GO enrichment analysis

GO annotations were obtained from the R package org.Mm.eg.db (version 3.4.0). For the enrichment of GO terms in the different gene lists, we only considered terms with 5–500 genes assigned to them. Significance of the enrichment was determined using Fisher's exact test with all expressed genes as background (Lackner et al. 2021). GO terms that did not differ in more than five genes were clustered, and one representative term for each cluster was defined (using the R base function hclust on the L1 distance of the binary membership matrix). As a representative GO term for each cluster, we chose the term with the lowest number of annotated genes. Multiple hypothesis testing was executed using the Benjamini–Hochberg method on all representative terms to calculate adjusted *P*-values.

### Quantification of mRNA half-lives

QuantSeq data were analyzed using SLAM-DUNK (version 0.2.4). For calculation of RNA half-lives, transcripts with CPM ≥ 2 were considered. Briefly, background (no 4SU treatment) was subtracted to calculate T > C conversion rates of transcripts of 4SU and 3- and 6-h uridine time points. Half-lives were calculated by plotting T > C conversion rates of 4SU and 3- and 6-h uridine as a single exponential fit to a decay model (Herzog et al. 2017).

### Exon conservation

Pairwise whole-genome alignments of mouse GRCm38/mm10 against dog canFam3, rat RGSC6.0/rn6, and human GRCh38/hg38 from UCSC (http://hgdownload.cse.ucsc.edu/downloads.html) were used for the analysis. With the Ensembl annotation of the mouse genome, we extracted the respective regions of the genes (introns, exons, and UTRs) and calculated their sequence identities compared with the other organism in the pairwise alignment (number of matching nucleotide pairs in the alignment) × 100/length). As gene region annotation can be ambiguous due to variations in transcript splicing, we used the following definitions: Everything that was annotated as coding sequence in any protein-coding transcript we counted as coding exon. UTR was everything that was annotated as UTR in any protein-coding transcript except if it was also annotated as coding sequence, in which case it was counted as coding exon. Regions not covered by the above definitions were counted as introns.

### RIP-seq analysis

Quality control of the transcripts from input and RIP samples was performed using FastQC. Reads were trimmed using bbduk (version 38.57). Reads were mapped to the mm10 mouse reference genome with STAR (version 2.5.3a). Afterward, indexing was performed using SAMtool (version 1.5), and reads in transcripts were counted with HTSeq-count (version 0.11.2). Transcripts that had CPM ≥ 1 in all the input samples were considered for the differential analysis. Differential analysis was performed using DESeq2 (version 1.24.0). To identify significantly enriched transcripts in eIF4A2^FL^ RIP, we considered only the 1231 transcripts with CPM ≥ 30 in eIF4A2^FL^ RIP and either log fold change eIF4A2^FL^ input/eIF42^FL^ RIP ≥ 1, RIP ≤ −1 (*P*adj ≤ 0.05), log fold change eIF4A2^FL^ RIP/EV RIP ≥ 1, or RIP/EV RIP ≤ −1 (*P*adj ≤ 0.05).

### Mass spectrometry data acquisition and analysis

The mass spectrometer was operated in data-dependent acquisition mode (DDA), and survey scans were obtained in a mass range of 375–1500 *m/z* with lock mass activated, at a resolution of 1.2 × 10^5^ at 200 *m/z* and an AGC target value of 3 × 10^6^. The eight most intense ions were selected with an isolation width of 1.6 *m/z* and fragmented in the HCD cell at 28% collision energy, and the spectra were recorded for a maximum of 250 msec at a target value of 1 × 10^5^ and a resolution of 3 × 10^4^. Peptides with a charge of +1 or more than +7 were excluded from fragmentation, the peptide match feature was set to preferred, the exclude isotope feature was enabled, and selected precursors were dynamically excluded from repeated sampling for 30 sec.

Raw data were processed using the MaxQuant software package (version 1.6.0.16) (Tyanova et al. 2016), the UniProt mouse reference proteome (January 2019, http://www.uniprot.org) and target sequences, and a database of the most common contaminants. The search was performed with full trypsin specificity and a maximum of two missed cleavages at a protein and peptide spectrum match false discovery rate of 1%. Carbamidomethylation of cysteine residues was set as fixed, and oxidation of methionine and N-terminal acetylation were set as variable modifications. For label-free quantification the “match between runs” feature and the LFQ function were activated; all other parameters were left at default.

MaxQuant output tables were further processed in R (https://www.R-project.org). Reverse database identifications, contaminant proteins, protein groups identified only by a modified peptide, protein groups with less than three quantitative values in one experimental group, and protein groups with less than two razor peptides were removed from further analysis. Due to differences in overall contaminant levels between samples, LFQ values were renormalized using the sample median of the “background” protein subset (as identified in controls). Missing values were replaced by randomly drawing data points from a normal distribution modeled on the whole data set (data mean shifted by −1.8 standard deviations, width of distribution of 0.3 standard deviations). Differences between groups were statistically evaluated using the limma package (Ritchie et al. 2015) at 5% FDR (Benjamini–Hochberg). The mass spectrometry proteomics data have been deposited to the ProteomeXchange Consortium via the PRIDE partner repository (Pérez-Riverol et al. 2019) under the accession number PXD019588. NGS data have been deposited on GEO (GSE153457).

## Supplementary Material

Supplemental Material
