# Supplementary material for: NMD is required for timely cell fate transitions by fine-tuning gene expression and regulating translation
Source: Genes Dev. 2022 Mar 1;36(5-6):348–67. doi: 10.1101/gad.347690.120 (PMC8973849; doi:10.1101/gad.347690.120)
Supplement: Supplemental Material [file supp_36_5-6_348__DC1.html]

NMD is required for timely cell fate transitions by fine-tuning gene expression and regulating translation — Supplemental Material 

# NMD is required for timely cell fate transitions by fine-tuning gene expression and regulating translation

## Supplemental Material

- Supplemental\_Table\_1\_Summary\_dKO\_generation.xlsx
- Supplemental\_Table\_2\_RNAseq.xlsx
- Supplemental\_Table\_3\_GO\_terms.xlsx
- Supplemental\_Table\_4\_Half\_life\_analysis.xlsx
- Supplemental\_Table\_5\_RIPseq.xlsx
- Supplemental\_Table\_6\_Mass\_spec.xlsx
- Supplemental\_Table\_7\_gRNAs\_and\_primers.xlsx
- Supplemental\_figures\_Huth\_347690.pdf
