# Supplementary material for: NMD is required for timely cell fate transitions by fine-tuning gene expression and regulating translation
Source: Genes Dev. 2022 Mar 1;36(5-6):348–67. doi: 10.1101/gad.347690.120 (PMC8973849; doi:10.1101/gad.347690.120)
Supplement: Supplemental Material [file supp_gad.347690.120_Supplemental_Information_.pdf]

Supplemental Information

NMD is required for timely cell fate transitions by fine-tuning gene expression and regulating translation

Michelle Huth,[1,13] Laura Santini,[1,2,13] Elena Galimberti,[1,13] Julia Ramesmayer,[1] Fabian Titz-Teixeira,[3] Robert Sehlke,[3] Michael Oberhuemer,[1] Sarah Stummer,[1] Veronika Herzog,[4] Marius Garmhausen,[3] Merrit Romeike,[1,2] Anastasia Chugunova,[5] Friederike Leesch,[2,5] Laurenz Holcik,[1,2,6] Klara Weipoltshammer,[7] Andreas Lackner,[1] Christian Schoefer,[7] Arndt von Haeseler,[1,6,8] Christa Buecker,[1] Andrea Pauli,[5] Stefan L. Ameres,[1,4] Austin Smith,[9,10] Andreas Beyer,[3,11,12] Martin Leeb[1]

Affiliations:

[1] Max Perutz Labs Vienna, University of Vienna, Vienna BioCenter, Dr.-Bohr-Gasse 9, 1030 Vienna, Austria

[2] Vienna BioCenter PhD Program, Doctoral School of the University of Vienna and Medical University of Vienna, 1030 Vienna, Austria

[3] Cluster of Excellence Cellular Stress Responses in Aging-associated Diseases (CECAD), University of Cologne, 50931 Cologne, Germany

[4] Institute of Molecular Biotechnology, Vienna BioCenter, Dr.-Bohr-Gasse 3, 1030 Vienna, Austria

[5] Research Institute of Molecular Pathology, Vienna BioCenter, Campus-Vienna-Biocenter 1, 1030 Vienna, Austria

[6] Center for Integrative Bioinformatics Vienna, Max Perutz Labs, University of Vienna and Medical University of Vienna, Dr.-Bohr-Gasse 9, 1030 Vienna, Austria

[7] Department for Cell and Developmental Biology, Medical University of Vienna, Schwarzspanierstrasse 17, 1090 Vienna, Austria

[8] Bioinformatics and Computational Biology, Faculty of Computer Science, University of Vienna, Vienna, Austria

[9] Wellcome - MRC Cambridge Stem Cell Institute, University of Cambridge, Cambridge CB2 0AW, UK

[10] Living Systems Institute, University of Exeter, Exeter EX4 4QD, UK

[11] Faculty of Medicine and University Hospital of Cologne, and Center for Molecular Medicine Cologne, University of Cologne, 50937 Cologne, Germany

[12] Institute for Genetics, Faculty of Mathematics and Natural Sciences, University of Cologne, 50923 Cologne, Germany

[13] These authors contributed equally to this work.

Corresponding author: martin.leeb@univie.ac.at

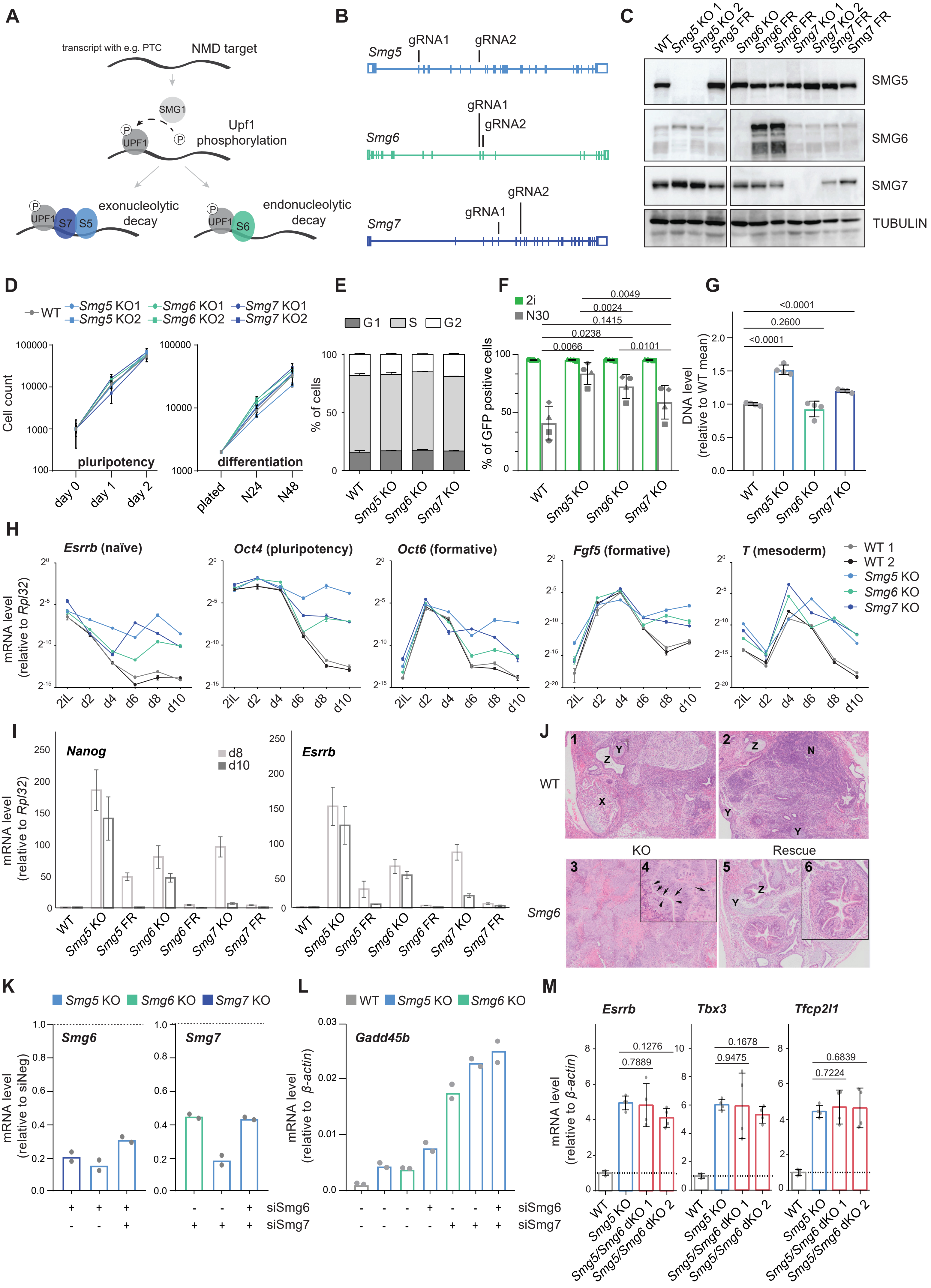

Supplemental Figure 1

Supplemental Fig. 1 | Hierarchy of defects in exit from naïve pluripotency in NMD-deficient ESCs. A, Illustration of the endo- and exonucleolytic branches of NMD, and involved key factors. **B**, Illustration of the strategy adopted to generate NMD KO ESCs utilizing CRISPR-Cas9. Gene structures of *Smg5*, *Smg6* and *Smg7* are shown. The position of each individual gRNA is indicated. **C,** Western blot analysis in WT, NMD KO and NMD rescue ESCs (Flag-rescue (FR)) using the indicated antibodies. TUBULIN was used as loading control. **D,** Growth curve of WT and NMD KO ESCs in naïve pluripotency supporting conditions (ES-DMEM + 2i, left panel) and during differentiation in N2B27 medium (right panel). Mean and standard deviation (SD) are plotted; n=3 biological replicates. Multiple t-tests were performed to compare WT to Smg-factor KO at each time point. Only *Smg6* KO2 and *Smg7* KO2 at N24 showed significantly different growth from WT cells. For all other comparisons, *p* values were > 0.05. **E,** Cell cycle analysis of WT and NMD KO ESCs. Propidium iodide was used to stain the cells and the Watson Pragmatic model was used to calculate the percentage of cells in each cell cycle phase. Mean and standard error of the mean (SEM) are plotted for each cell cycle phase; n=3 biological replicates. No statistically significant aberrations were detected by an ANOVA test. **F,** Quantification of Rex-GFPd2 positive cell populations in WT and *Smg*-factor KO ESCs; n=4 biological replicates. Paired, two-tailed t-test was performed. *p* values are indicated in the plot. **G,** Telomere length analysis in WT and NMD KO ESCs. Results were normalized to the 36B4 single copy gene. Mean and SD of n=4 technical replicates are plotted for each cell line. Unpaired, two-tailed t-test was performed to compare WT to NMD KO ESCs. *p* values are indicated in the plot. **H,** Expression kinetics of the indicated genes during a 10-day embryoid body (EB) differentiation assay measured by RT-qPCR. Mean and SD of technical replicates are plotted for each time point. Expression levels were normalized to *Rpl32.* The assay was performed twice. One representative experiment is shown. **I,** Rescue cell lines analysed in an EB differentiation time course at day 8 and 10 of differentiation by RT-qPCR. *Nanog* and *Esrrb* expression is shown. Normalisation was performed against *Rpl32,* and expression levels are shown relative to WT. Mean and standard error of the mean (SEM) for n=2 biological replicates are shown. **J,** Teratomas derived from WT, *Smg6* KO and *Smg6* rescue ESCs. **1-2**: areas of WT-derived teratoma showing well differentiated tissues of mesodermal (e.g., X enchondral ossification; Y cartilage), endodermal (e.g., Z), and ectodermal (neuronal rosettes; N) origin. **3**: Area of *Smg6* KO-derived teratomas showing abundant poorly differentiated neuronal tissue; poorly differentiated endodermal and mesodermal tissues are present at lower abundancy (not shown). Abundant mitotic structures present in neuronal tissue (arrows) demonstrate high proliferative capacity (**4**). **5**: Area of *Smg6* rescue-derived teratomas showing examples of well differentiated endodermal (Z) and mesodermal (Y: cartilage) tissues. Higher power magnification shows an endodermal duct with different, well developed epithelial cell types (**6**). **K,** RT-qPCR analysis confirming the siRNA-mediated knockdown of NMD components. Mean and

SD of technical replicates are plotted for each cell line. Expression was normalized to *Rpl32*; results are shown as expression levels relative to siNeg (dashed line). **L,** Expression levels of *Gadd45b* in the indicated cell lines after siRNA-treatment measured by RT-qPCR. Expression was normalized to β-a*ctin*. Mean and SD of replicates are plotted for each cell line (n=2 biological replicates). **M**, RT-qPCR analysis of the indicated genes at N24 in WT, *Smg5* KO and *Smg5/Smg6* dKO cell lines. Expression was normalized to β-a*ctin*; results are shown as expression levels relative to WT. Mean and SD are plotted; n=2 biological replicates. A paired, two-tailed t-test was performed to compare *Smg5* KO with *Smg5/Smg6* dKOs. *p* values are indicated in the plot.

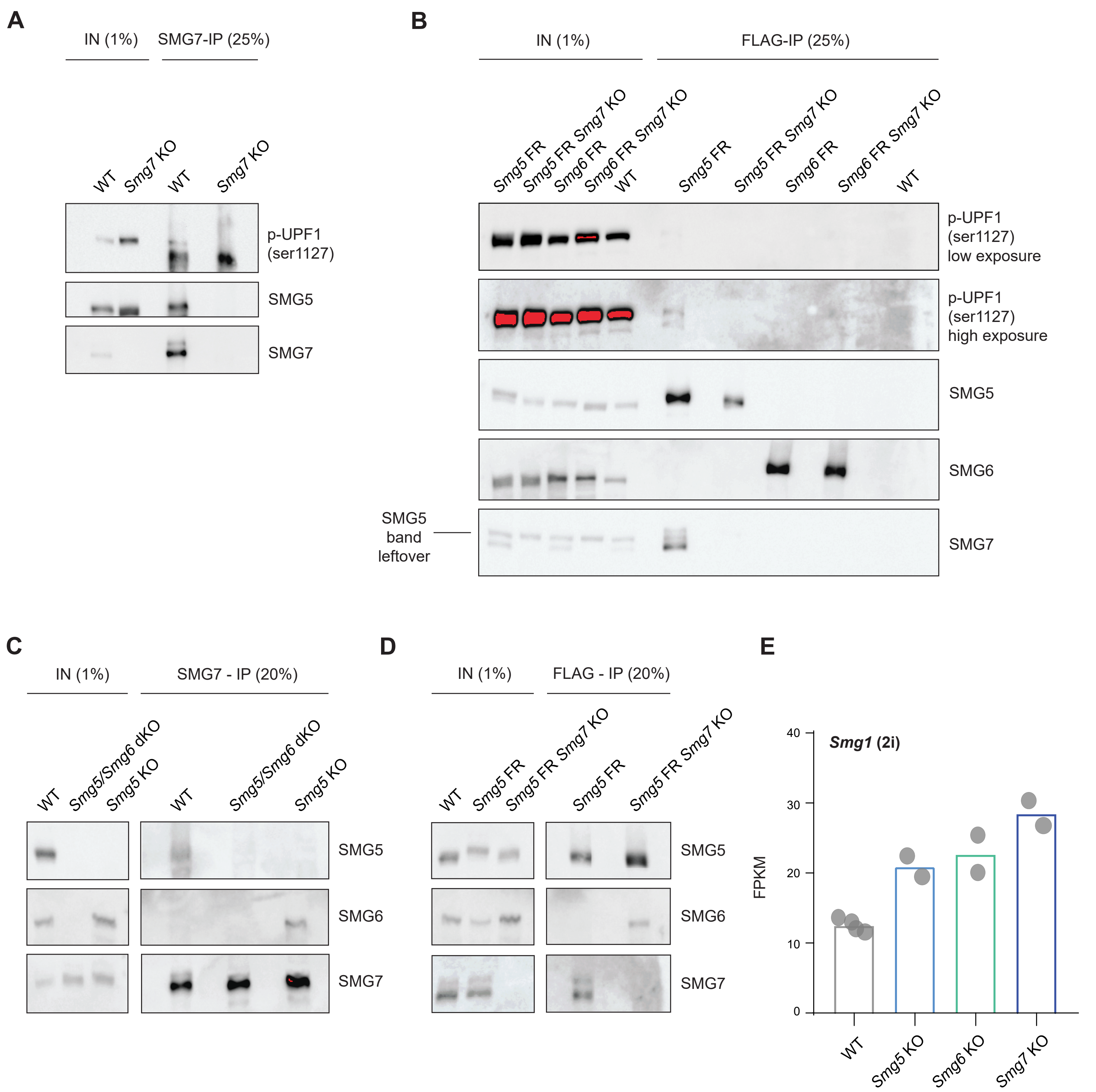

Supplemental Figure 2

Supplemental Fig. 2| SMG7 alone is sufficient for p-UPF1 binding and required for mediating the SMG5-p-UPF1 interaction. A, Western blot analysis of SMG7 co-IP in the indicated cell lines without cyanase treatment. Antibodies used are indicated in the Figure. **B,** Western blot analysis of SMG5 co-IP (flag antibody co-IP) in the indicated cell lines without cyanase treatment. Antibodies used are indicated in the figure. A and B show control experiments for the Western blots shown in Figures 2A and 2B. There is no unspecific co-precipitation by the antibodies used. **C,** Western blot analysis after SMG7 co-IP in the indicated cell lines without cyanase treatment. Antibodies used are indicated in the figure. **D,** Western blot analysis of SMG5 co-IP (flag antibody co-IP) in the indicated cell lines without cyanase treatment. Antibodies used are indicated in the figure. C and D show experiments performed in parallel to those shown in Figures 2A and 2B and show one representative of two experiments with consistent results. **E,** Expression levels (FPKM) of *Smg1* derived from RNA-Seq analysis in WT and NMD KO ESCs in 2i. Mean values between biological replicates are plotted.

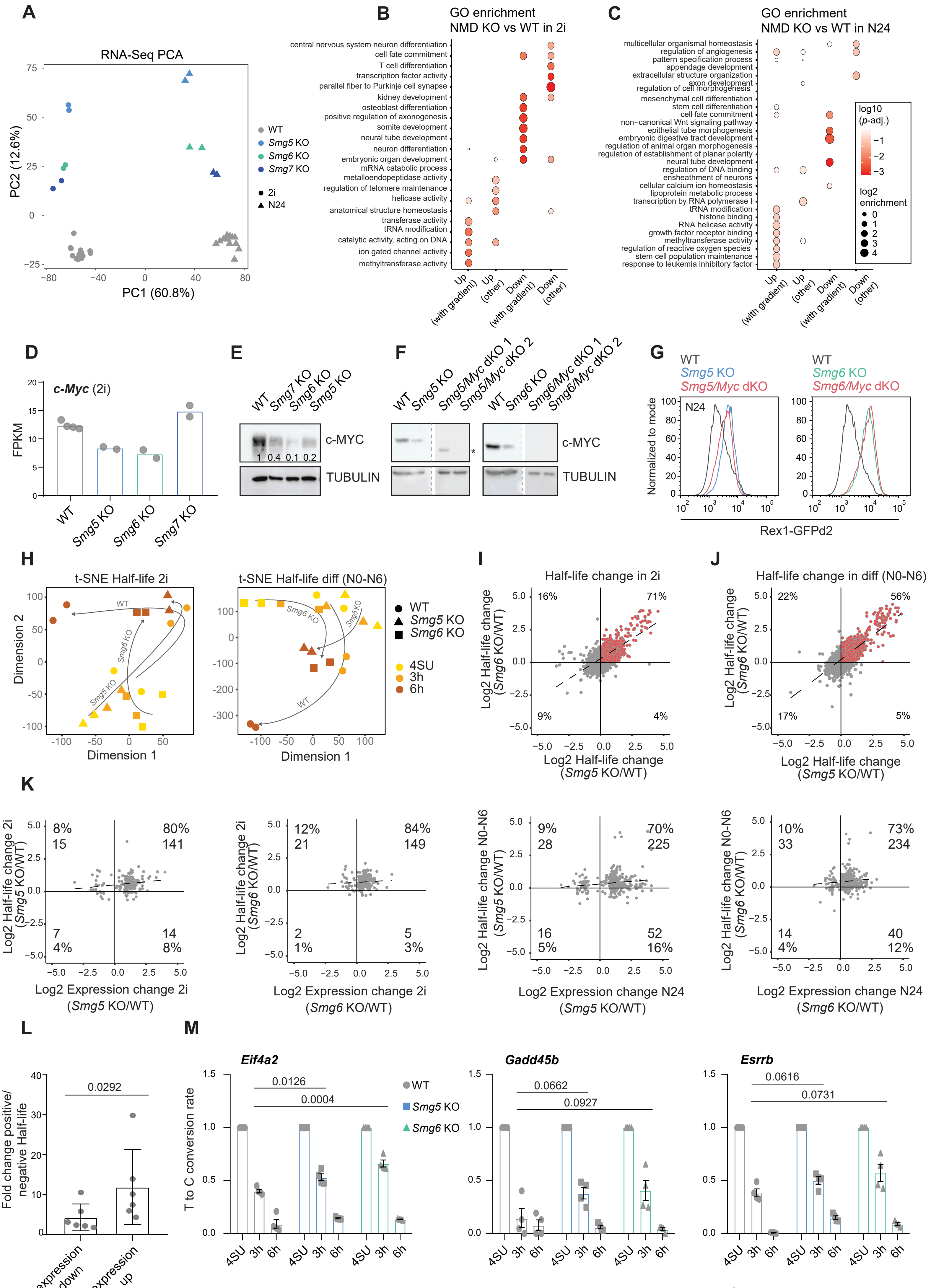

Supplemental Figure 3

Supplemental Fig. 3 | Integrating transcriptome-wide gene expression with mRNA half-life analysis identifies relevant NMD targets during the exit from naïve pluripotency. A, PCA plot based on RNA-Seq data of WT and Smg-factor KO ESCs in 2i and 24h after 2i withdrawal (N24). Each symbol indicates one specific sample as indicated in the legend. **B,** Significantly enriched GO terms of differentially expressed transcripts in Smg-factor KOs in 2i, as indicated on the x-axis. **C,** Similar to B for differentially expressed transcripts at N24. **D,** Expression levels (FPKM) of *c-Myc,* derived from RNA-Seq in WT and Smg-factor KO ESCs in 2i. Mean values between biological replicates are plotted. Statistical analysis between WT and Smg-factor KO ESCs showed no significant increase in *c-Myc* expression levels. **E,** Western blot analysis for c-MYC expression in WT and NMD KO ESCs. TUBULIN was used as loading control. **F,** Western blot analysis confirming absence of c-MYC protein in *Smg5* or *Smg6*/*c-Myc* dKO ESCs. TUBULIN was used as loading control. * band corresponds to a potential truncated protein. **G,** Rex1-GFPd2 flow cytometry profiles at N24 in WT, Smg-factor KO and *Smg5* or *Smg6*/*c-Myc* dKO cells. **H,** t-SNE plot showing SLAM-Seq data. Colors indicate timepoints and symbols indicate genotypes. Arrows were manually added and show the trajectory during the chase. **I,** Comparison of half-life (HL) changes between *Smg5* and *Smg6* KO cells in 2i. **J,** As in I for HL changes at the onset of differentiation (N0-N6). **K,** Correlation between differential expression and HL changes. All genes which are significantly deregulated in 2i or at N24 in RNA-Seq for which half-lives could be calculated are plotted. The first two panels show a comparison between HL changes in 2i and expression changes in 2i for *Smg5* KOs (first) and *Smg6* KO (second). The third and fourth panels show a comparison between HL changes at the onset of differentiation (N0-N6) for *Smg5* KOs (third) and *Smg6* KO (fourth) and expression changes at N24. **L,** Plot showing quantification of correlation of HL and transcription. The ratio between positive and negative HL changes is plotted for upregulated and downregulated transcripts. Each dot corresponds to one comparison shown in K, additionally including the comparison between HL changes in 2i and expression changes at N24. Mean and SD are indicated in the graph. A paired t-test was used to calculate *p* values. **M,** Quantification of T to C conversion rates on selected targets in WT, *Smg5* and *Smg6* mutant cell lines throughout a 4SU-based pulse chase experiment (SLAM-Seq). Mean and SD are indicated in the graph. Unpaired t-test was used to calculate *p* values for the 3h timepoint. Data obtained in 2i and at N0-N6 were merged as *bona-fide* replicates in these plots.

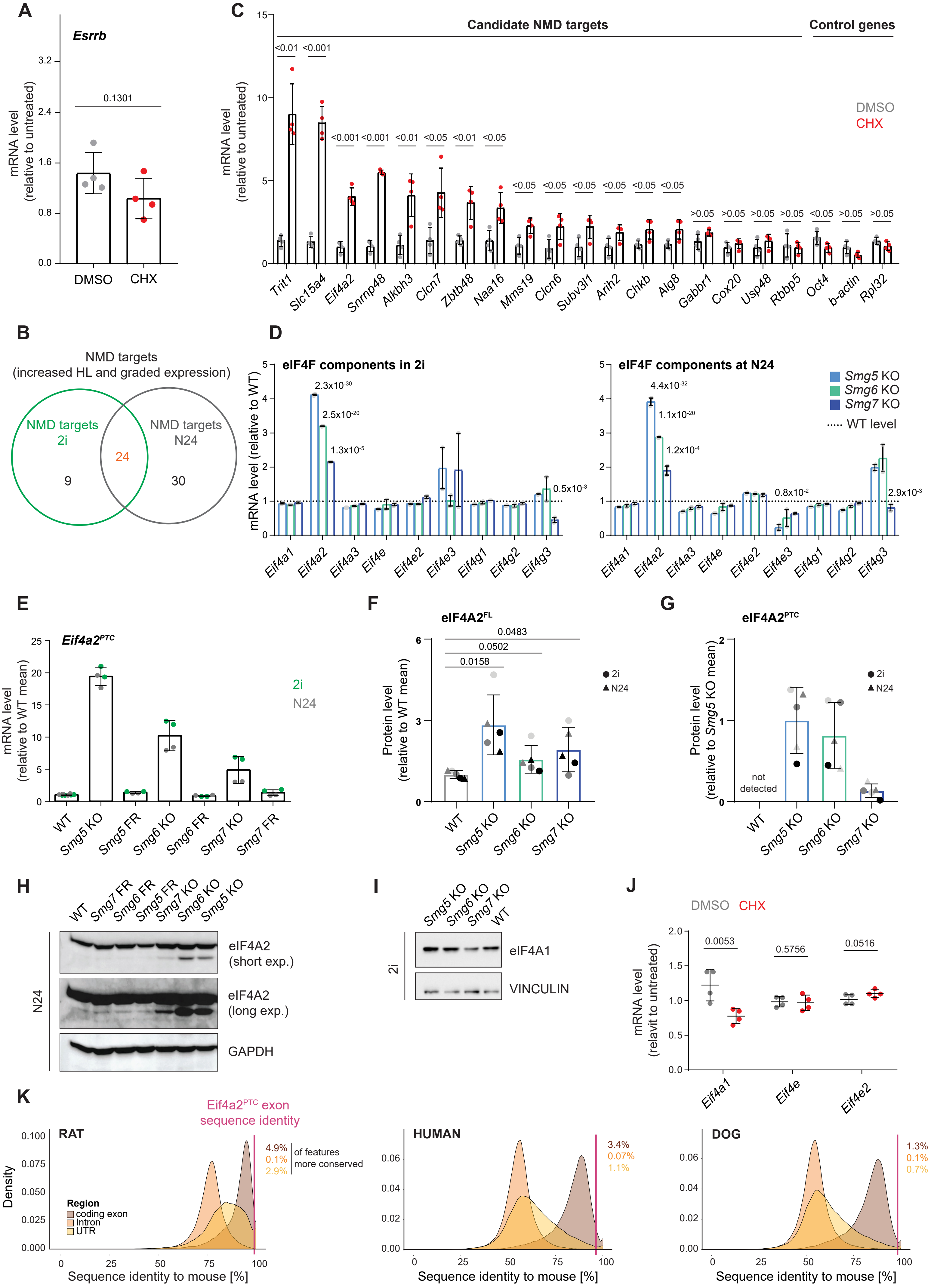

Supplemental Figure 4

Supplemental Fig. 4 | *Eif4a2* is a *bona-fide* NMD target in ESCs. A, RT-qPCR results showing *Esrrb* expression after treatment with cycloheximide (CHX) or DMSO in WT ESCs. Data shown is normalized to untreated WT ESCs. Mean and SD are plotted; n=4 biological replicates. Unpaired, two-tailed t-test was performed to compare DMSO and CHX treatments and the resulting *p* value is shown in the plot. **B**, Overlap of transcripts belonging to the 'Increased HL in 2i and at N0-N6 in *Smg5* and *Smg6* KOs' group (see Fig. 3F) and showing graded upregulation either in 2i ('NMD targets 2i', green) or at N24 ('NMD targets N24', grey). **C**, Transcript levels of indicated candidate NMD targets (18 out of 24 candidates identified in B) and controls after treatment with DMSO or CHX in WT ESCs measured by RT-qPCR. Data shown is normalized to untreated WT ESCs. Mean and SD are plotted; n=4 biological replicates. Unpaired, two-tailed t-test was performed between DMSO and CHX comparison for each gene, and the resulting *p* values are shown in the plot. **D**, Expression level (based on FPKM derived from RNA-Seq analysis) of eIF4F complex components in Smg-factor KO ESCs relative to WT ESCs in self-renewal (2i, left) and at N24 (right). Mean and SEM of biological duplicates are plotted. Statistically significantly differential expression following a graded pattern was only detected for total *Eif4a2* (including all transcript variants). *p* values are indicated in the plots. **E**, RT-qPCR data showing expression levels of *Eif4a2*^{PTC} in WT, Smg-factor KO and NMD rescue cell lines in 2i and at N24. Expression levels were normalized to *Rpl32*; results are shown as expression levels relative to the WT mean. Mean and SD for n=2 technical replicates for each time point are shown. **F**, Quantification of eIF4A2^{FL} protein expression in WT and Smg-factor KO ESCs in 2i and at N24 measured by Western blot analysis. Expression was normalized to VINCULIN; results are shown as expression levels relative to the WT mean. Mean and SD for n=3 (2i) and n=2 (N24) independent experiments (distinguished by different grayscale colors) are shown. Paired, two-tailed t-tests were performed to compare WT with each Smg-factor KO. *p* values are indicated in the plot. **G**, Quantification of eIF4A2^{PTC} protein expression in WT and NMD KO ESCs in 2i and at N24 measured by Western blot analysis. Expression was normalized to VINCULIN; results are shown as expression levels relative to the *Smg5* KO mean. Mean and SD for n=3 (2i) and n=2 (N24) independent experiments (distinguished by different grayscale colors) are shown. **H,** Western blot analysis for eIF4A2 protein levels in WT, NMD KO and NMD rescue ESCs (Flag-rescue (FR)) at N24. GAPDH was used as loading control. **I,** Western blot analysis for eIF4A1 expression in WT and NMD KO ESCs. VINCULIN was used as loading control. **J,** Expression levels of the indicated genes measured by RT-qPCR after treatment with CHX or DMSO in WT ESCs. Data shown is normalized to untreated WT ESCs. Mean and SD are plotted; n=4 biological replicates. Unpaired, two-tailed t-test was performed between DMSO and CHX comparison for each gene, and the resulting *p* values are shown in the plot. **K**, Densities of the distribution of the sequence identities of indicated regions of the mouse transcriptome compared to rat, human and dog. The pink line indicates the level

of sequence identity of the PTC-containing *Eif4a2* exon. Percentage of genomic features with higher conservation than the *Eif4a2 PTC*-exon are indicated in the graphs.

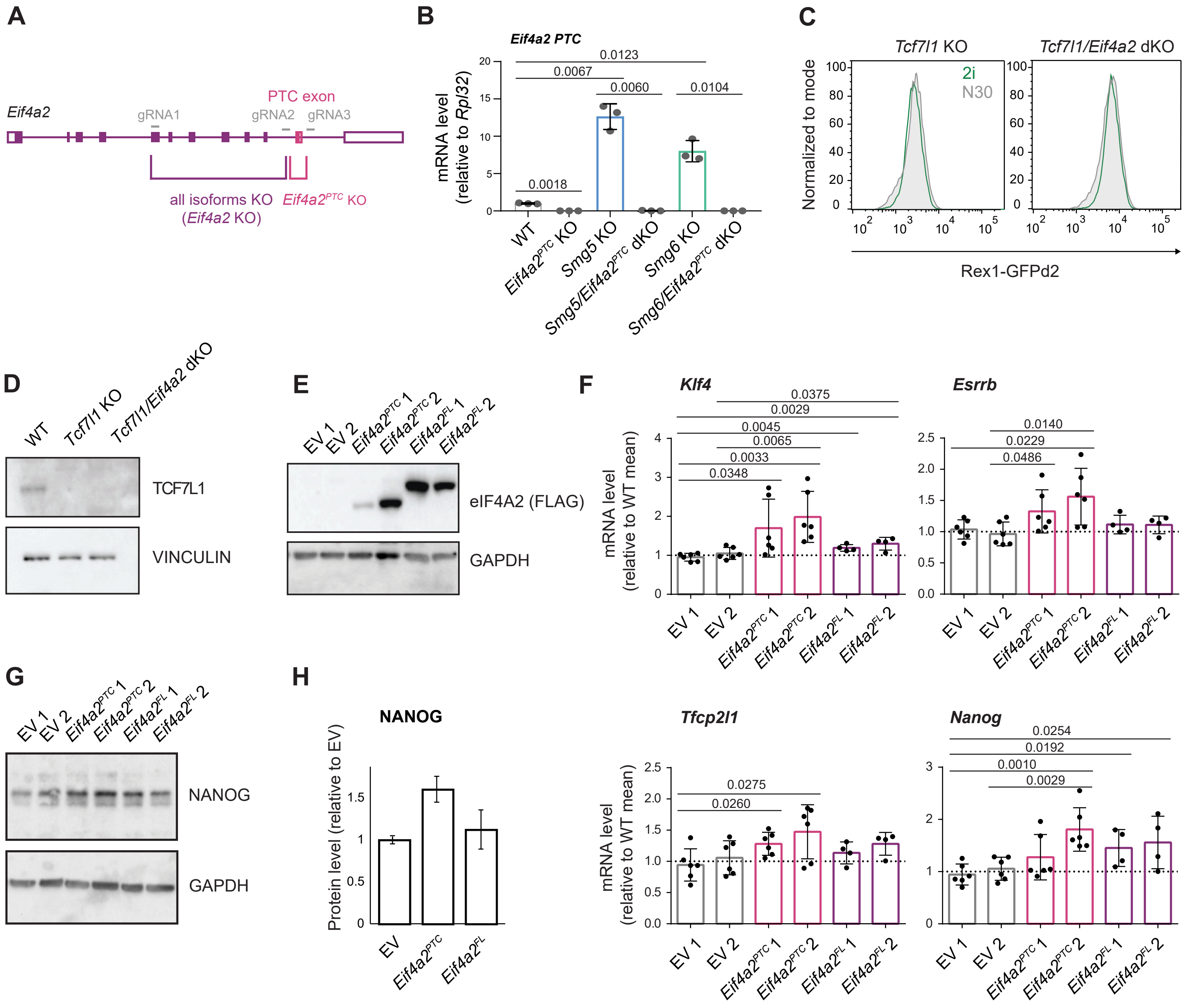

Supplemental Figure 5

Supplemental Fig. 5 | *Eif4a2* is causative for defects in exit from naïve pluripotency in *Smg5*-deficient ESCs. A, Illustration of the strategy to generate *Eif4a2* and *Eif4a2^{PTC}* KO ESCs utilizing CRISPR-Cas9. The *Eif4a2* gene structure is shown, and the position of each individual gRNA is indicated. gRNA1 and gRNA3 were used in combination to delete all *Eif4a2* isoforms. gRNA2 and gRNA3 were used in combination to delete the *Eif4a2^{PTC}* isoform. **B,** RT-qPCR using *Eif4a2^{PTC}*-specific primers. Expression was normalized to *Rpl32*. Mean and SD for n=3 independent experiments are shown. Paired, two-tailed t-test was used to calculate *p* values. **C,** Rex1-GFPd2 flow cytometry profile in *Tcf7l1* KO and *Tcf7l1/Eif4a2* dKO ESCs in 2i (green) and at N30 (grey). **D,** Western blot analysis for TCF7L1 expression in the indicated cell lines. VINCULIN was used as loading control. **E,** Western blot analysis showing eIF4A2^{FL} and eIF4A2^{PTC} protein levels upon overexpression of 3xFLAG-eIF4A2^{FL} or 3xFLAG-eIF4A2^{PTC} in WT. WT cells transfected with an empty vector (EV) show no signal. GAPDH was used as loading control. **F,** RT-qPCR analysis of the indicated genes at N30 in EV, eIF4A2^{FL}- and eIF4A2^{PTC}-overexpressing cell lines shown in E. Expression was normalized to *Rpl32*; results are shown as expression levels relative to untransfected WT cells (dotted line). Mean and SD are plotted for each cell line, n=3 biological replicates. Unpaired, two-tailed t-test was used to calculate *p* values. **G,** Western blot analysis for NANOG expression in the indicated cell lines at N24. GAPDH was used as loading control. **H,** Bar graph showing quantification of NANOG protein levels in eIF4A2^{FL}- and eIF4A2^{PTC}- overexpressing cell lines relative to EV, normalized to GAPDH. Mean and SEM are shown.

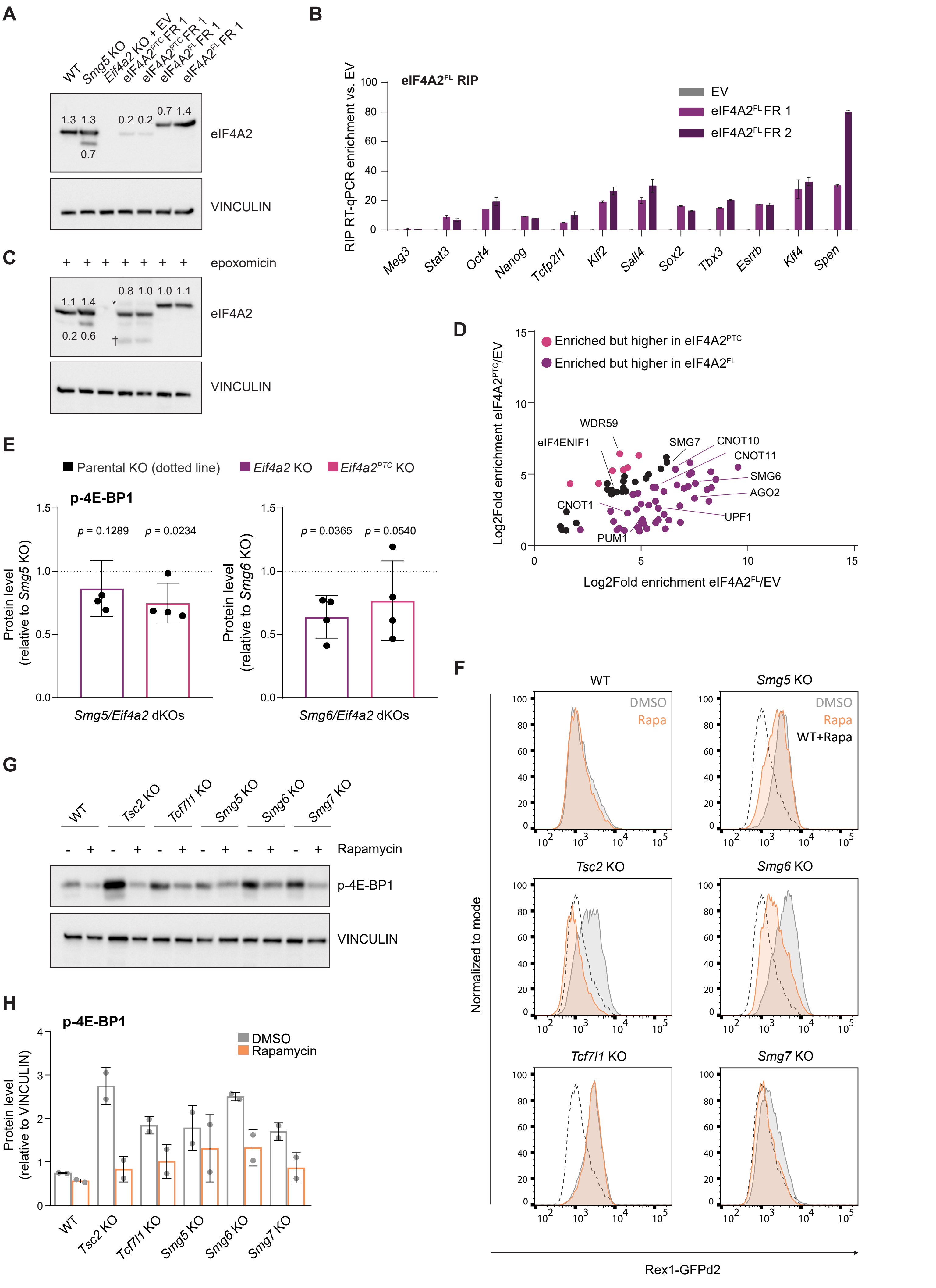

Supplemental Figure 6

Supplemental Fig. 6 | *Eif4a2*-mediated differentiation delay is caused by PTC isoform-dependent regulation of translation A, Western blot analysis for eIF4A2 in clonal *Eif4a2* KO cells in which $eIF4A2^{PTC}$ or $eIF4A2^{FL}$ isoforms were re-expressed from transposon-based vectors (2 clones each, used as biological replicates in mass-spectrometry and RIP experiments). VINCULIN was used as loading control. Numbers in the plot indicate protein levels relative to VINCULIN. **B,** RT-qPCR validation of $eIF4A2^{FL}$ RIP-Seq experiments**.** The non-protein-coding *Meg3* gene was used as negative control. Values were normalized to empty vector (EV)-RIP and to input. Mean and SD are plotted. Biological duplicates of $eIF4A2^{FL}$ RIP-samples are shown separately in shades of purple. RIP-Seq results were confirmed utilizing primers detecting a set of pluripotency marker genes. *Spen* serves as positive control and was one of the most enriched transcripts in RIP-Seq analysis. **C,** Western blot analysis as in A, but after treatment with epoxomicin. Numbers in the plot indicate protein levels relative to VINCULIN. † indicates potential degradation products, and * refers to potential post-translational modifications**. D,** Dot-plot showing mass-spectrometry enrichment in $eIF4A2^{PTC}$ and $eIF4A2^{FL}$ co-IP in ESCs. Only proteins that were detected as interactors of both eIF4A2 isoforms are plotted. Proteins more strongly bound to $eIF4A2^{PTC}$ are depicted as pink dots. Proteins more strongly bound to $eIF4A2^{FL}$ are depicted as purple dots. **E**, Quantification of p-4E-BP1 protein levels in 2i in *Smg5* KO (left) and *Smg6* KO (right) ESCs depleted for *Eif4a2* or $Eif4a2^{PTC}$, measured by Western blot analysis. Expression levels were normalized to GAPDH; results are shown as expression levels relative to the respective parental cell line (dotted line). Mean and SD for n=4 independent experiments are shown. Paired, one-tailed t-tests were performed to compare Smg-factor KO ESCs deficient for *Eif4a2* or $Eif4a2^{PTC}$ with their parental Smg-factor KO. *p* values are indicated in the plot. **F,** Rex1-GFPd2 flow cytometry analysis of the indicated cell lines treated with DMSO (grey) or with 20 nM Rapamycin (Rapa, orange). Rex1-GFPd2 levels of Rapamycin-treated WT cells are shown as black dashed line. One representative of n=5 independent experiments is shown. **G,** Western blot analysis for p-4E-BP1 expression in the indicated cell lines, upon treatment with DMSO (-) or 20 nM Rapamycin (+). VINCULIN was used as loading control. One of n=2 independent experiments is shown. **H,** Bar graph showing quantification of p-4E-BP1 protein expression measured by Western blot analysis (normalized to VINCULIN) in n=2 independent experiments. SEM is shown.
